# Structure-Based Design of Inhibitors of Protein–Protein Interactions: Mimicking Peptide Binding Epitopes

**DOI:** 10.1002/anie.201412070

**Published:** 2015-06-26

**Authors:** Marta Pelay-Gimeno, Adrian Glas, Oliver Koch, Tom N Grossmann

**Affiliations:** Chemical Genomics Centre of the Max Planck Society Otto-Hahn-Strasse 15, 44227 Dortmund (Germany) E-mail: tom.grossmann@cgc.mpg.de; TU Dortmund University, Department of Chemistry and Chemical Biology Otto-Hahn-Strasse 6, 44227 Dortmund (Germany)

**Keywords:** inhibitors, peptides, peptidomimetics, protein–protein interactions

## Abstract

Protein–protein interactions (PPIs) are involved at all levels of cellular organization, thus making the development of PPI inhibitors extremely valuable. The identification of selective inhibitors is challenging because of the shallow and extended nature of PPI interfaces. Inhibitors can be obtained by mimicking peptide binding epitopes in their bioactive conformation. For this purpose, several strategies have been evolved to enable a projection of side chain functionalities in analogy to peptide secondary structures, thereby yielding molecules that are generally referred to as peptidomimetics. Herein, we introduce a new classification of peptidomimetics (classes A–D) that enables a clear assignment of available approaches. Based on this classification, the Review summarizes strategies that have been applied for the structure-based design of PPI inhibitors through stabilizing or mimicking turns, β-sheets, and helices.

## 1. Introduction

**Figure 1:**
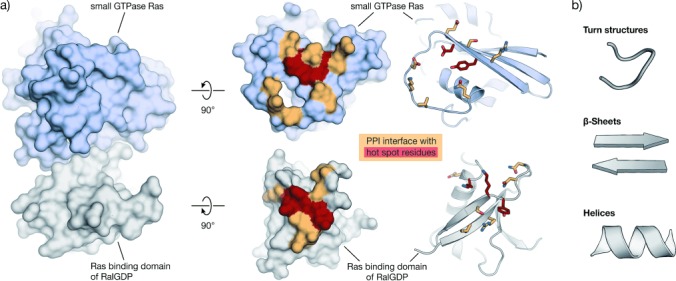
Example of a PPI with its interaction area and hot spots: a) Left: Crystal structure in surface representation of the complex between Ras (light blue) and the Ras binding domain of RalGDP (gray, PDB: 1LFD).[5] Right: The same proteins with their PPI interface (orange/red). Hot-spot residues are highlighted in red. Proteins are shown in surface and schematic representation (interacting residues are shown as sticks);[6] b) Peptide secondary structures in schematic representation.

Protein–protein interactions (PPIs) are involved in most cellular processes and influence biological functions through proximity-induced changes of protein characteristics, such as enzymatic activity, subcellular localization, and/or binding properties. Therefore, the modulation of PPIs is considered a promising strategy towards next-generation therapeutics.[1] In contrast to small molecular ligands that bind to defined protein pockets, the interfaces of PPIs often involve rather flat protein surfaces that exhibit an average area of 800–2000 Å^2^.[2] PPIs can occur between two structured protein domains, a structured domain and a relatively short peptide, or between two peptide stretches. In many cases, additional weak contacts distant to the defined interaction area contribute to binding, thereby adding complexity and complicating the prediction of PPI characteristics.[3] However, the investigation of numerous PPI interfaces (orange/red, Figure 1 a) revealed that certain protein side chains mainly contribute to the Gibbs energy of protein–protein binding. These so-called hot-spot residues (red, Figure 1 a) often overlap with structurally conserved regions and represent a common feature of PPI interfaces.[2, 4]

Modulators of PPIs are important for the elucidation of biological processes and are considered promising candidates in drug development.[1, 7, 8] In many cases, small molecular scaffolds used in standard drug design failed to provide active and selective PPI inhibitors. This is not surprising, given the large and often shallow interaction areas of PPIs and the fact that most small molecular drugs target well-defined cavities of enzymes or receptors. Consequently, the chemical space of traditional small-molecule libraries deviates from that of PPI inhibitors, thus leading to low hit rates when applied in screening for PPI inhibitors.[9] This stimulated the search for alternative strategies involving fragment-based screens or natural product inspired libraries that contain molecules with relatively high molecular weights and a large number of stereocenters.[1] Additionally, computational tools have been used to design more diverse sets of compounds or to perform in silico screens with improved virtual libraries.[10–12] In a structure-based approach, peptide binding epitopes derived from protein interaction sites can serve as a starting point for the design of PPI inhibitors. Such epitopes are defined by the secondary structure of the underlying polypeptide chain that aligns amino acid side chains in a defined manner. Depending on the backbone conformation, secondary structure elements can be grouped into regular β strands and helices with specific, repetitive torsion angle ranges,[13–15] and into nonrepetitive irregular turn structures or loops that show a wide range of torsion angles (Figure 1 b).[16, 17] Importantly, peptides tend to lose their secondary structure when excised from the stabilizing context of their protein domain and exist in an ensemble of conformational states when free in solution. This flexible nature renders peptides prone to proteolytic degradation and results in relatively low target affinity as a result of entropic penalties upon binding.[18]

**Figure 2:**
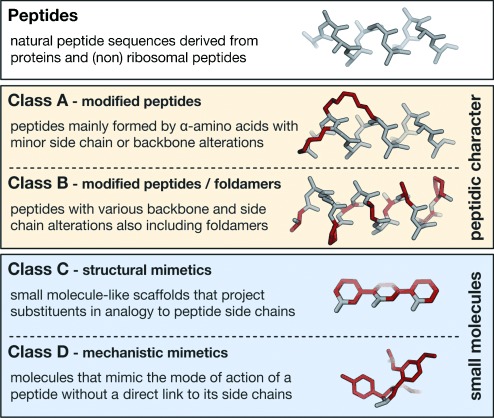
Classification of peptidomimetics used in this Review: For illustration, an α-helical peptide and corresponding helix mimetics are shown. Modifications are highlighted in red.

Efficient mimicking of peptides in their bioactive conformation is a long-standing goal in chemical sciences and not only related to the design of PPI inhibitors. Several strategies have been evolved to enable a projection of side chain functionalities, in analogy to peptide secondary structures, to yield molecules that are generally referred to as peptidomimetics.[19] Peptidomimetics are defined inconsistently in the literature: ranging from very narrow definitions that only cover molecular scaffolds replacing the peptide backbone, to broader definitions that also include modified peptide sequences with improved biological properties.[20–24] In this Review we will use a broad definition of peptidomimetics that covers all the designed molecules that mimic the binding properties of natural peptide precursors. Historically, type I mimetics are defined as short peptides that replicate the topography of a secondary structure. These mimetics distinguish themselves from their parent peptide only by substitutions introduced to stabilize the desired conformation (in many cases an α-helix). Type II mimetics refer to functional mimetics that have a small molecular scaffold and do not necessarily recapitulate all the side chain interactions of the parent protein. Finally, type III mimetics include nonpeptide templates that are topologically similar to the parent peptide but do not show atom-by-atom analogy.[20, 21] This historic classification of peptidomimetics does not comply with recent advances in the field nor does it allow clear assignment of all approaches. In addition, it insufficiently visualizes the degree of abstraction relative to the parent peptide. For these reasons, we here introduce a new classification of peptidomimetics based on the degree of their similarity to the natural peptide precursor, thereby resulting in four different classes A–D, where A features the most and D the least similarities (Figure 2). Classes A and B include peptide-like structures (differentiating type I and partially including type III mimetics) whereas classes C and D encompass small molecular scaffolds (including type II and to some extent type III mimetics):

Marta Pelay Gimeno completed her BSc in chemistry at the University of Barcelona. She then joined the group of Prof. F. Albericio at the Institute for Research in Biomedicine (IRB) in Barcelona, where she obtained her PhD in organic chemistry in 2013 for the synthesis and structure elucidation of biologically active marine peptides. Currently she is a postdoctoral fellow with Dr. T. N. Grossmann, where she applies her knowledge of peptide chemistry towards strategies that enable the stabilization of peptide and protein structures.

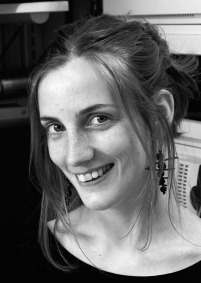

Adrian Glas was born in Berlin, Germany, and studied chemistry at the Humboldt University, receiving his diploma in 2011. In 2012 he joined the group of Dr. T. N. Grossmann as a PhD student, where his work focuses on the stabilization of irregular peptide secondary structures and their application as inhibitors of protein–protein interactions.

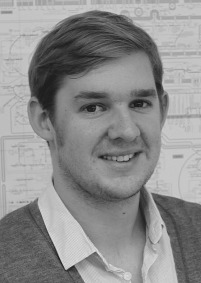

Oliver Koch studied pharmacy and computer science at the Philipps-University Marburg, Germany, where he also obtained his PhD in pharmaceutical chemistry with Prof. G. Klebe. After postdoctoral research at the Cambridge Crystallographic Data Centre in 2008 and working in drug discovery at MSD Animal Health Innovation, he started his independent academic career in 2012 as a group leader for medicinal chemistry at the Technical University Dortmund, Germany. His research interests involve the development and application of computational methods in rational drug design.

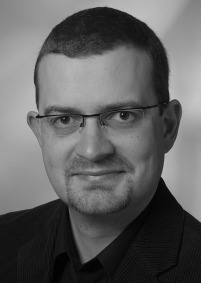

Tom N. Grossmann studied chemistry at the Humboldt University Berlin, Germany. After undergraduate research with K. P. C. Vollhardt at the University of California Berkeley, he received his PhD with Prof. O. Seitz at the Humboldt University Berlin in 2008. He then became a postdoctoral researcher in the group of Prof. G. L. Verdine at Harvard University. Thereafter, he became group leader at the Technical University and the Chemical Genomics Centre in Dortmund, Germany. His research interests involve the stabilization of peptide secondary structures and the development of biocompatible reactions.

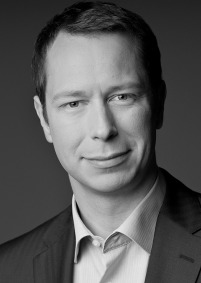

- Class A mimetics are defined as peptides that mainly consist of the parent peptide amino acid sequence. Only a limited number of modified amino acids are introduced to stabilize the bioactive conformation. The backbone and side chains of a class A mimetic align closely with the bioactive conformation of the precursor peptide.
- Class B involves further modified class A mimetics with various non-natural amino acids, isolated small-molecule building blocks, and/or major backbone alterations. This class also includes foldamers, such as β- and α/β-peptides as well as peptoids, which align their side chains topologically similar to the precursor peptide.
- Class C includes highly modified structures with small-molecule character that replace the peptide backbone completely. The central scaffold projects substituents in analogy to the orientation of key residues (e.g. hot spots) in the bioactive conformation of the parent peptide.
- Class D mimetics are molecules that mimic the mode of action of a bioactive peptide without a direct link to its side chain functionalities. Such molecules can be generated by affinity optimization of a class C molecule or they can be identified in screenings of compound libraries or by in silico screening of virtual libraries.

This Review summarizes strategies that were applied for the structure-based design of PPI inhibitors using peptide binding epitopes as the starting point in the design process. For this reason, scaffolds derived from screenings of small-molecule or peptide libraries were not included. We focus on mimicking approaches that do not significantly increase the complexity and size of the inhibitor, thereby excluding strategies such as the grafting of binding epitopes on miniproteins or peptide toxins.[1] Interactions that are mediated by posttranslational modifications such as phosphorylation or lipidation are also not discussed, as these are mainly driven by the recognition of the modification. The Review is divided into two sections: The methodology part (Section 2) describes general approaches towards mimicking peptide binding epitopes, with a focus on strategies that have been used to design PPI inhibitors. Section 3 describes the application of these methods to a variety of biologically relevant PPIs, focusing on well-established model systems.

## 2. Methodology

### 2.1. Mimetics of Turn Structures

**Figure 3:**
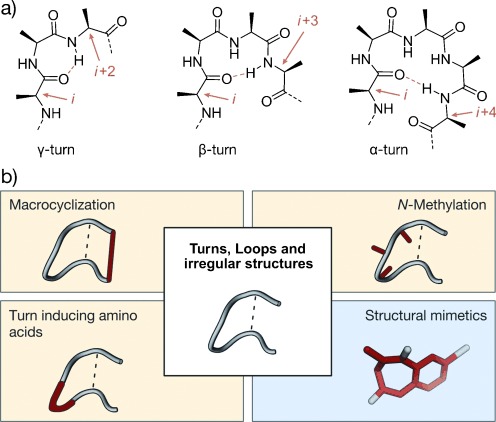
Turns with general stabilization and mimicking approaches: a) Chemical structure of a γ-, β-, and α-turn; stabilizing hydrogen bonds are indicated by dotted orange lines, participating residues by orange arrows. b) General strategies for turn stabilization and mimicry (highlighted in red; class A and B: yellow; class C: blue).

With a length of two to six amino acids, turns are irregular secondary structure elements that differ from helices and β-sheets through the nonrepetitive dihedral angles of their backbones. The term loop is often used as a synonym, but throughout this Review loops comprise the irregular part of a polypeptide chain outside of helices and β-strands.[25, 26] Historically, turn structures were defined as regions that allow a polypeptide chain to fold back on itself, thereby enabling the formation of globular proteins.[27] Over the last few decades, several more general definitions were described, with a widely used one classifying turns in accordance to the hydrogen-bond pattern formed between the backbone carbonyl group of the residue at position *i* and the backbone amide proton at position *i*+*n*.[28] This leads to the four families of γ-, β-, α-, and π-turns with three to six amino acids in length and *n*=2, 3, 4, and 5, respectively (Figure 3 a). A reverse hydrogen-bonding pattern is observed between the main chain amide proton at position *i* and the carbonyl group at position *i*+n for δ- and ε-turns with *n*=1 and 2, respectively.[29] In addition, similar backbone conformations can occur that lack a hydrogen bond but show a specific Cα_*i*_–Cα_*i*+*n*_ distance, so-called open turns.[30, 31] Within a turn family, subgroups or turn types can be defined on the basis of different backbone conformations and the dihedral angles *ϕ* and *ψ* involved. Since their first analysis by Venkatachalam,[27] definitions for β-turn types were adjusted several times,[32–34] finally leading to the widely used nine β-turn types defined by Hutchinson and Thornton: types I, I′, II, II′, VIa1, VIa2, VIb, VIII, and IV.[30] Likewise, similar analyses were carried out for the remaining turn families. Recently, an analysis of turn backbone conformations in available protein structures led to a uniform classification of all turn families.[17, 35] Taking into consideration that there is a high occurrence of nonrepetitive turn regions in weak and transient heterodimers,[36] this classification may provide the rationale towards novel PPI inhibitors. In addition to single turn conformations, there are so-called turn motifs which involve overlapping turn structures.[37, 38] Although not yet analyzed in detail in the context of PPIs, turn motifs frequently occur in structured protein domains, in particular in loop regions which are considered important in PPIs.[39]

Mimicking the conformation of PPI-relevant turn structures is considered a promising strategy towards PPI inhibitors. For peptide-derived mimetics, certain backbone conformations can be enforced by macrocyclization, turn-inducing amino acids, and N-methylation (Figure 3 b), thereby yielding class A and B mimetics. The combinations of these approaches can increase the stabilizing effect and is often required for the development of high affine binders. An alternative strategy that yields structural mimetics (class C) involves the use of small molecular scaffolds that replace the entire peptide backbone and align side chains in a spatial arrangement according to the peptide turn residues. Although turns play an important role in PPIs and turn mimetics appear to be a promising approach for the design of corresponding inhibitors, only a few examples have been reported so far. Most examples involve inhibitors of enzymes (e.g. proteases) or of interactions between peptide ligands and proteins (e.g. ligand-activated G-protein-coupled receptors). To discuss the underlying concept of turn mimetics, we will highlight some examples of inhibitors of ligand–protein interactions. First, we will introduce approaches that aim to mimic single-turn structures, which is followed by mimetics of turn motifs.

#### 2.1.1. Single-Turn Mimetics

##### 2.1.1.1 Macrocyclization

In natural peptides and proteins, macrocyclization frequently occurs as a constraining element in turn structures, for example through disulfide or thioether bridges.[40, 41] Inspired by this, numerous cyclization strategies have been reported over the last decades, including head to tail, side chain to backbone, and side chain to side chain cyclizations.[42, 43] Early examples of designed macrocyclic peptides as turn mimetics have been described for sequences derived from peptide ligands that target membrane-associated receptors. Pioneering work in this field was performed by Kessler and co-workers, who intensively investigated the structure–activity relationship of head to tail cyclized peptides by NMR spectroscopy.[44] The impact of N-methylation and epimerization of the amino acids involved in the conformational flexibility of penta- and hexameric cyclic peptides as PPI inhibitors was studied. By using disulfide cross-linked natural peptides as inspiration, Grubbs and co-workers were able to replace the disulfide by hydrocarbon cross-links and conserve the initial bioactive conformation.[45] Another naturally inspired cyclization strategy to afford bioactive PPI inhibitors involves the incorporation of binding motifs into the so-called cysteine ladders, which appear in θ-defensins as a parallel arrangement of disulfide bonds that stabilize a turn structure.[46] Alternatively, peptide epitopes have been grafted onto lasso peptides, thus allowing their preorganization into bioactive conformations.[47] These genetically encoded peptides form a macrocycle with their C-terminal tail passing through this ring system. This conformation is usually locked by bulky side chains.[48] Recently, an approach towards bicyclic peptides by cross-linking thiol-containing amino acids was introduced. Either three natural cysteines can be cross-linked by a trifunctional molecule to form stable thioether bonds or a non-natural dithiol bearing amino acid can form two disulfide bridges to two native cysteines.[49, 50] So far, these two strategies have not been applied for a structure-based design of PPI inhibitors. However, given the conformational rigidity and structural diversity of these scaffolds, their successful application as PPI inhibitors can be anticipated.

##### 2.1.1.2. Turn-Inducing Amino Acids

Since cyclization alone is often insufficient to ensure the population of a single conformation, further restraining elements are applied. In γ-turns, for example, sterically demanding residues are often observed at position *i*+1.[17, 27] Other amino acids such as Pro, Gly, Asn, and Asp are overpopulated in β-turns.[16, 17] Proline, the only proteogenic amino acid with a secondary backbone amine and a ring structure involving backbone atoms, plays a unique role in protein folding. This is due to a reduced conformational flexibility and the absence of the amide proton, which prevents hydrogen-bond formation. Additionally, the secondary amine introduces a bulkier substituent, thereby driving the *cis*–*trans* equilibrium of the amide bond towards the *cis* isomer.[51] Furthermore, d-amino acids at position *i*+1 proved useful as β-turn inducers,[52–54] and d-proline (d-Pro or p) as a stabilizer of β-turns.[55–57] Kessler and co-workers performed a spatial screening on cyclic pentapeptides to analyze the influence of d-amino acids. Starting with an all-l-peptide, one amino acid at a time was substituted by its d enantiomer.[58] The most stable βII′/γ conformation involved a d-amino acid at position *i*+1 of the βII′-turn. In addition, nonproteinogenic amino acids are used to promote turn conformations involving, for example, sugar amino acids (SAAs).[59] Another frequently applied amino acid is α-aminoisobutyric acid (Aib), which is known to reduce conformational freedom and induce β-turns.[60] Since β-hairpins consist of two β-strands connected by a turn structure, turn-inducing amino acids are also valuable scaffolds to stabilize β-sheets (Section 2.2.1).

##### 2.1.1.3. N-Methylation

In proteins, N-methylation only occurs as a side chain modification. However, N-methylation of the backbone is observed frequently in nonribosomal natural peptides, especially from marine or fungal origin. A prominent example of a natural cyclic peptide used as an orally administered therapeutic is cyclosporine A, which appears to be heavily N-methylated.[61] In general, the methylation of the amide nitrogen atom can have several effects on the structural properties of peptides:[62] N-methylation alters the hydrogen-bond pattern by reducing the number of hydrogen-bond donors. Additionally, N-methylation influences the *cis–trans* equilibrium of the amide bond, as observed for proline, thus rendering the *cis* less unfavored. Furthermore, the increase in steric hindrance affects the conformational freedom of the adjacent amino acids.[63] Through these effects, N-methylation can influence the overall backbone conformation. N-Methylation proved useful as a constraining element, especially in short cyclic peptides. Kessler and co-workers studied the conformations of 30 cyclic model peptides. All peptides were head to tail cyclized pentaalanines, containing four l- and one d-amino acid combined with mono-, di-, tri-, or tetra-N-methylation. It was observed that seven of these peptides exist in only a single conformation with six of them being methylated at the d-amino acid. This is in agreement with the observation that N-methylation can mimic proline, which is known to stabilize β-turns in its d configuration.[55–57] In addition, nine peptides of this library adopt several conformations, with one conformation being overpopulated by more than 80 %.[64, 65] The identification of general design principles is complicated, since the influence of modifications highly depends on the sequence context, and minor conformational changes, especially in PPIs, can have significant effects on the affinity and selectivity. Importantly, N-methylation also contributes to increased protease resistance, which is further enhanced when combined with macrocyclization and the introduction of unnatural amino acids.[62] Overall, these features can yield highly potent bioactive molecules as described for the cyclic pentapeptide and clinical candidate Cilengitide.[66–68]

##### 2.1.1.4. Structural Mimetics

**Figure 4:**
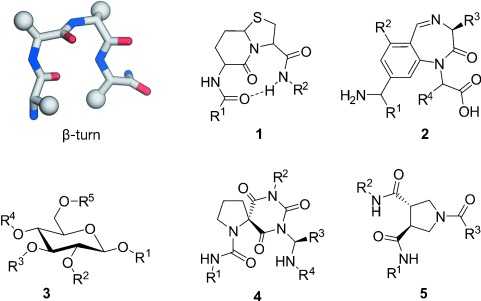
Scaffolds that mimic β-turn conformations: Bicyclic peptide (1),[71] benzodiazepine (2),[75] glucose (3),[77] spirocyclic mimetic (4),[82] and *trans*-pyrollidine-3,4-dicarboxamide (5).[83]

The replacement of the entire peptide backbone in a turn structure by small-molecule scaffolds is an alternative approach that can result in molecules with improved oral bio-availability and pharmacokinetic properties. Notably, the resulting bioactive compounds are more likely to follow Lipinski′s rule of five, thus rendering them promising candidates in drug development.[69] Small-molecule scaffolds that mimic turn structures should align their substituents in a spatial arrangement similar to the side chains in the corresponding turn type. Many of these class C mimetics were designed on the basis of structural comparison with turn conformations. Successful examples mainly involve the design of inhibitors of interactions between peptide ligands and proteins. For example, a subset of G-protein-coupled receptors (GPCRs) recognizes peptide ligands that contain turn structures[70] and have been mimicked using small molecules. Here, we illustrate the general concept of structural-turn mimetics based on findings derived from these investigations. It remains to be seen if they are also applicable for the design of PPI inhibitors. As a consequence of their frequent occurrence and thorough characterization, β-turns (Figure 4, left) are prime targets for structural mimetics (class C), with so-called bicyclic turned peptide **1** being one of the first examples (Figure 4).[71] Nagai and Sato showed that this bicyclic compound has the backbone conformation of d-Ala-l-Pro in Gramicidin S, with a type II′ β-turn. These findings were the basis for several other constrained bicyclic scaffolds with different ring sizes that mimic β-turns.[72–74] Benzodiazepines (**2**, Figure 4) are the most prominent examples of a turn mimetic.[75] As a consequence of the two distinct conformations of its central seven-membered ring, the scaffold can be used to mimic almost all β-turn types.[76] Glucose is another scaffold (**3**, Figure 4) that has been used to mimic β-turns, with a focus on mimicking the cyclic peptide somatostatin.[77] Notably, tetrahydropyrane-based β-turn mimetics were included in peptide sequences to serve as PPI inhibitors.[78] An alternative class of β-turn mimetics are spirocyclic compounds (**4**, Figure 4) initially described by Robinson and co-workers[79] and analyzed in detail by Gmeiner and co-workers.[80–82] Recently, *trans*-pyrollidine-3,4-dicarboxamide (**5**, Figure 4) was shown to mimic β-turns that harbor proline at position *i*+1.[83] Notably, Müller et al. re-analyzed some of the described turn mimetics in detail and suggested that not all categorized scaffolds recapitulate the anticipated turn structures correctly.[84]

#### 2.1.2. Turn Motif Mimetics

Turn motifs are defined as overlapping turn conformations such as the Shellman motif,[37, 85] in which a hydrogen-bonded type I β-turn is encapsulated by a hydrogen-bonded type I π-turn that serves as a capping motif,[86] and as specific turns such as the Asx motif in which an aspartate or asparagine side chain at position *i* interacts with a backbone amide at position *i*+2.[87] Although already described decades ago,[38, 88] overlapping turns have not been thoroughly investigated, especially in the context of mimicking approaches. Given the importance of extended turn structures in PPIs[36, 39] and the possibility of their computational analysis,[17, 89] the search for turn-motif mimetics holds the potential to provide novel classes of PPI inhibitors. As an early example, Gellman and co-workers reported the replacement of α- by β-amino acids in a phage display derived peptide[90] that harbors hot-spot residues in the last turn of an α-helix and in the following turn structure.[91] The final peptide with increased protease resistance indicates the general possibility of turn-motif mimetics. So far, the sole example of a stabilized natural turn motif used as a PPI inhibitor was described by Grossmann and co-workers.[92, 93] They designed a small library of constrained peptides based on the crystal structure of a human adaptor protein and a bacterial virulence factor. Hydrophobic residues, which have been reported to be crucial for the interaction, were chosen to be substituted by an all-hydrocarbon cross-link. The optimization of the linker length and configuration led to a macrocyclic peptide that was capable of inhibiting the interaction between the two binding partners in vitro. The large diversity of turn motifs and their intense engagement in protein interactions complicates the identification of general design principals which may explain the small number of approaches for their mimicry.

### 2.2. Mimetics of β-Strands and β-Sheets

**Figure 5:**
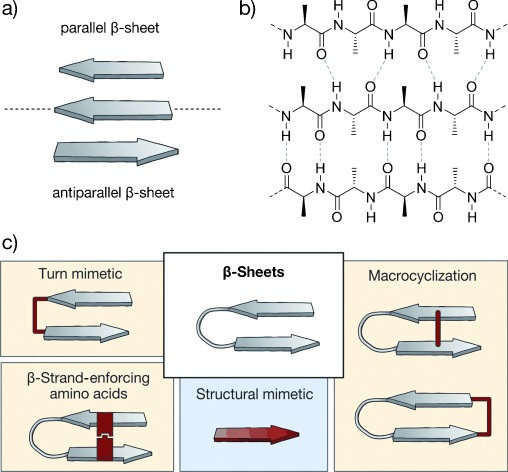
β-Sheets with general stabilization and mimicking approaches: a) Schematic representation of parallel (top) and antiparallel (bottom) β-sheets. b) Chemical structure of a parallel (top) and antiparallel (below) β-sheet arrangement. Hydrogen bonds are represented by dashed lines. c) General strategies to afford β-sheet mimetics (highlighted in red; class A and B: yellow; class C: blue).

β-Strands are structural elements in which the peptide adopts an extended conformation with well-defined dihedral angles that arrange the amide bonds almost coplanar and the side chains alternatively above and below this plane. Notably, hydrogen bonds are only formed between β-strands and not within a strand, thereby supporting β-sheet formation by parallel or antiparallel alignment of multiple β-strands (Figure 5 a,b). β-Sheets are highly involved in the formation of tertiary as well as quaternary protein structures, protein aggregation, and protein–protein interactions. The combination of two antiparallel β-strands connected through a turn provides a β-hairpin which is stabilized by an extended pattern of interstrand hydrogen bonds.[94] As a result of their straightforward accessibility by solid-phase peptide synthesis, the folding properties of these structures have been studied extensively,[95] which fostered access to β-hairpin mimetics.

Several methods have been developed to synthesize modified β-strands, β-hairpins, and β-sheets.[96–98] Three general approaches to yield class A and B β-sheet mimetics can be distinguished: The use of turn mimetics that nucleate β-sheet formation, covalent or noncovalent macrocyclization (backbone or side chain to side chain), and the use of β-strand-enforcing amino acids (Figure 5 c, yellow). In many cases, the intrinsic complexity of the β-sheet secondary structure requires the combination of these approaches to ensure appropriate stabilization of the β-sheet. In addition, several small-molecule structural mimetics of β-strands (class C) have been reported (Figure 5 c, blue). Their suitable functionalization and diversification is a challenge, and strategies for the construction of complex structural mimetics of β-sheets remain elusive. In general, there are very few examples of β-sheet mimetics described as PPI inhibitors. However, the large number of approaches developed for the stabilization and mimicry of β-sheets and their application as inhibitors of, for example, proteases,[99] indicate a possible use of these approaches for the design of PPI inhibitors. Consequently, we will also highlight examples that have not so far been used in PPI inhibition.

#### 2.2.1. Stabilized β-Sheets

##### 2.2.1.1. Turn Mimetics as β-Hairpin Inducers

**Figure 6:**
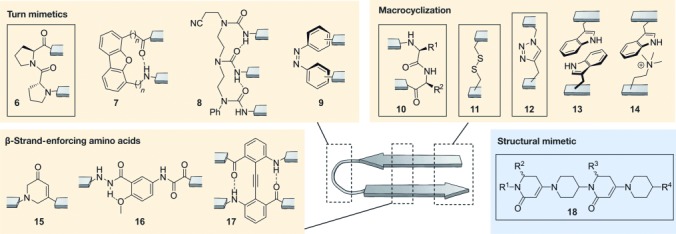
β-Sheet mimetics: Turn mimetics: l-Pro-d-Pro (6), dibenzofuran derivatives (7), oligourea (8), azobenzene (9); Macrocyclization: head to tail (10), side chain to side chain cross-link with a disulfide (11) and 1,2,3-triazole ring (12), side chain to side chain π–π interaction (Trp–Trp; 13) and cation–π interaction (*N*^δ^-trimethylornithine–Trp; 14); β-strand-enforcing amino acids: 1,6-dihydro-3(2*H*)-pyridinone (Ach, 15), Hao building block (16), diphenylacetylene building block (17); Structural mimetics: piperidine–piperidinone-based strand mimetic (18); class A/B: yellow; class C: blue. Box: structure used in the context of PPI inhibition.

A number of turn mimetics that effectively induce the formation of β-hairpins have been designed (Figure 6), often in analogy to previously described turn mimetics (Section 2.1.1). d-Amino acids at turn position *i*+1 were used to promote type II′ β-turns, thereby supporting β-hairpin formation.[56, 100] Furthermore, the presence of N-alkylated amino acids and a combination of prolines and aromatic residues in the turn region enabled efficient nucleation of β-hairpins.[101] Thus, templates such as d-Pro-l-Pro (**6**)[102–104] and d-Pro-Gly[57, 105, 106] are privileged dipeptides used extensively to stabilize antiparallel β-hairpins, while d-Pro-DADME (1,2-diamino-1,1-dimethylethane) provides parallel β-sheet arrangements.[107, 108] Other dipeptides such as Aib-Gly (where Aib is 2-aminoisobutyric acid)[109] and Asn-Gly, although less efficient, have also been used to induce β-sheet conformations.[110, 111] Turns based on di-β-peptides also proved useful in the nucleation of β-hairpins in both β-[112] and α-peptides.[113] So far, d-Pro-l-Pro (**6**) is the only turn mimetic that has been employed in the stabilization of β-hairpin-based PPI inhibitors (in combination with macrocyclization, see Section 2.2.1.2).[114, 115]

A variety of small molecular scaffolds represents well-suited structural alternatives for peptidic-turn mimetics, but without applications in PPI inhibitors so far. Examples involve dibenzofuran derivatives (**7**),[116, 117] oligoureas (**8**),[118–120] azobenzenes (**9**),[121] and others.[122–128] Assisted by their hydrogen-bond pattern, oligoureas (**8**) can nucleate β-hairpins as well as more-complex β-sheet scaffolds. Notably, azobenzenes (**9**) enable the light-induced control of β-hairpin formation. Moreover, some examples of metal-directed β-sheet formation have been reported. Several complexes of copper,[129, 130] ruthenium,[131] iron,[132] zinc,[130, 133] and platinum[134] serve as nucleating motifs for parallel and antiparallel β-sheet structures.

##### 2.2.1.2. Macrocyclization

In nature, macrocyclic β-hairpins fulfill a variety of biological functions. An illustrative example is the Tachyplesin family, which comprises potent antimicrobial peptides isolated from hemocytes of horseshoe crab[135] that proved useful as inhibitors of HIV replication.[136] Tachyplesins are amphiphatic peptides containing two disulfide bridges that lock the antiparallel β-hairpin conformation.[137] The presence of these disulfide bonds in natural products underlines the relevance of macrocyclization for the stabilization of isolated β-hairpin structures. Seminal research has been conducted with Tachyplesin peptides, thereby providing a number of simplified analogues,[138–140] in some cases with modified scaffolds.[141] In analogy to constrained naturally occurring β-hairpins, covalent and noncovalent macrocyclization approaches have been applied to stabilize β-sheet arrangements. Approaches based on covalent macrocyclization and noncovalent capping motifs proved efficient (Figure 6) in reducing the terminal fraying typically featured by β-hairpins. Covalent macrocyclization, mainly head to tail (**10**), has been used extensively to avoid terminal unfolding and to reinforce interstrand hydrogen bonding.[142–144] In addition, noncovalent capping motifs have been evaluated, mainly based on electrostatic interactions,[145–147] with one exception based on hydrophobic contacts.[148] Andersen and co-workers described a capping motif consisting of an N-terminal acetylated Trp and a Trp-Thr-Gly sequence at the C-terminus.[149] A face to edge interaction between the two indoles, and hydrogen bonds between polar groups in Thr, Gly, Trp, and the alkanoyl group are responsible for the stabilization.

Covalent and noncovalent macrocyclization can also occur through side chains along the strands of a β-hairpin. Thus, disulfide bridges (**11**) between cysteines located at amino acid positions not involved in hydrogen bonding result in hairpin stabilization and can be used to construct stable[150–152] and more-complex quaternary β-sheet structures.[153] Another covalent interaction used to stabilize β-hairpins is a 1,2,3-triazole moiety (**12**) formed by means of click chemistry[154–156] which has recently been employed to synthesize a PPI inhibitor.[157] Alternatively, tryptophan zippers (**13**) have been shown to also contribute to β-hairpin stabilization.[158–160] To favor β-hairpin formation, the tryptophan moieties must be located at amino acid positions that are not involved in hydrogen-bond formation, with the greatest stabilizing effect when placed in proximity to the β-turn. In contrast, other hydrophobic interactions such as Phe-Phe pairs require a modification of hydrogen-bonded sites. Tryptophan residues also participate in cation–π interactions (**14**) between side chains. The stabilizing effect of this cross-strand pairing strongly depends on the chain length and degree of methylation of the basic amino acid.[161–163]

##### 2.2.1.3. β-Strand-Enforcing Amino Acids

A highly prolific strategy to induce and stabilize β-sheet structures is the use of conformationally constrained building blocks that reproduce the typical hydrogen-bonding pattern of a β-sheet, thereby acting as β-strand mimics (Figure 6). Examples include 1,6-dihydro-3(2 H)-pyridinone (Ach, **15**),[164] the unnatural amino acid Hao (5-hydrazino-2-methoxybenzoic acid, **16**),[165] and diphenylacetylene residue **17**,[166] among others.[167] Bartlett and co-workers first described the use of Ach (**15**) for the stabilization of β-strands and β-hairpins. This building block can be further functionalized[168] and used in solid-phase syntheses (SPS) to access oligomeric compounds.[169] Analogously, Hao (**16**) and its derivatives[170] are rigid amino acids that mimic the pattern of hydrogen-bond donors and acceptors in one β-strand, thus enabling the construction of well-folded β-sheet structures. Hao (**16**), described by Nowick et al., has been frequently used in combination with oligourea templates (**8**)[171] or ornithine amino acids that act as δ-sided turns.[172] Moreover, Hao (**16**) and other moieties also proved useful for the initiation of intermolecular β-sheet formation.[173, 174] Hamilton and co-workers used a diphenylacetylene moiety (**17**) to arrange two strands in an antiparallel β-sheet.[166] This moiety concomitantly replaces one residue in each strand, thereby reproducing the distinctive interstrand hydrogen bonds of a β-sheet structure and incorporating a covalent cross-link between the two strands. Notably, the use of β-amino acids to reinforce the β-sheet secondary structure proved to be extremely challenging due to a profound mismatch of structural features between α- and β-peptides. Single replacements mostly result in partially unfolded structures,[175] and α,α-dipeptide substitutions provided only moderate results.[176] So far, none of the mimics with β-strand-reinforcing amino acids have been described as PPI inhibitors.

#### 2.2.2. Structural β-Strand Mimetics

β-Sheet integrity heavily relies on hydrogen-bond-mediated interactions between amino acids with large spacing in the primary sequence, which complicates the design of self-assembled β-sheet structures. This also holds true for the development of structural β-sheet mimetics using small-molecule scaffolds. So far, only the β-strand—the basic unit of β-sheet structures—has been suitably mimicked (Figure 6). Only a few examples have been reported, including the 1,3-substituted triazole oligomers published by Angelo and Arora,[177] the 2,2-disubstituted-indolin-3-ones from Wyrembak and Hamilton,[178] the pyrrolinone-based scaffolds described by Smith et al.,[179] and the piperidine-piperidinone-based molecules (**18**) reported by Burguess and co-workers.[180] Notably, the last examples have been used as PPI inhibitors; they are chiral structures with limited rotational freedom that lack enolizable positions and are accessible by facile and modular synthesis. Interestingly, this scaffold was designed to mimic both β-strand and helical conformations.

### 2.3. Mimetics of Helices

**Figure 7:**
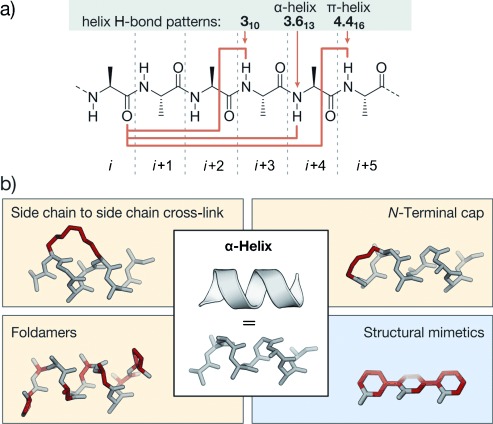
Helices with corresponding stabilization and mimicking approaches: a) Chemical structure of a peptide chain, helix-stabilizing hydrogen-bond patterns are indicated by orange arrows. b) Schematic representation of an α-helix together with general strategies of helix stabilization and mimicry (highlighted in red; class A and B: yellow; class C: blue).

Helices are repetitive secondary structure elements which make up more than 30–40 % of structured protein domains.[89] They are stabilized by intramolecular hydrogen bonds between sequential residues (*i* and *i*+*n*; Figure 7 a). A general nomenclature for helices uses the number of residues participating in one turn of the helix and gives the number of atoms in the ring formed by the hydrogen bond between the carbonyl group of the amino acid at position *i* and the amide proton at position *i*+*n* in subscript.[181, 182] In natural proteins, only helices with an integral number between three and five are observed (3_10_-, 3.6_13_-, and 4.4_16_-helix), even if others are theoretically stable.[13] Only the 3_10_-helix, which consists of repetitive β-turns, retained its nomenclature. The 3.6_13_- and 4.4_16_-helices, built up by consecutive α- and π-turns, are better known as α- and π-helices, respectively. Whereas the π-helix is rarely observed in protein secondary structures,[183, 184] 3_10_-helices contribute to 10 % of all helical regions in globular proteins.[185] The remaining 90 % are α-helices. Based on available structural data,[186] helices contribute to the protein–protein interface in 62 % of all PPIs,[187] thus highlighting the importance of α-helices in this context. Different stabilization approaches have been reported since the early 1980s.[188] Most prominent strategies towards the preparation of class A mimetics involve side chain to side chain cross-linking of peptides and the introduction of stabilizing N-terminal caps (Figure 7 b). The use of foldamers represents an alternative approach towards the synthesis of class B helix mimetics. Foldamers are peptide and nucleic acid inspired oligomers which exhibit major backbone alterations. In addition, several structural mimetics (class C) have been reported. These scaffolds include rodlike templates capable of projecting substituents in analogy to certain side chains of an α-helix.

#### 2.3.1. Side Chain to Side Chain Cross-Links

α-Helices are stabilized by intramolecular hydrogen bonds between the carbonyl oxygen atom and the amide proton at positions *i* and *i*+4, respectively. Additional stabilization may occur through the formation of salt bridges between residues (e.g. glutamic acid and lysine) that are aligned on the same face of a helix. This stabilizing effect has been utilized in early examples of class A mimetics that aimed to stabilize the helical conformation.[189] Later, covalent cross-links incorporated at positions *i*, *i*+3, or *i*, *i*+4 were applied to bridge one turn of a helix, and at positions *i*, *i*+7 for two helical turns. Early examples of covalent cross-links include the formation of an amide bond between glutamic acid and lysine[190] and the assembly of disulfide bonds between cysteine analogues.[191] The combination of side chain cross-linking and helicity enforcing α-carbon methylation[188] led to a technique called hydrocarbon peptide stapling.[192] These peptides bear an all-hydrocarbon cross-link formed by ring-closing metathesis (RCM). Alternatively, α-helices have been stabilized by transition-metal-mediated or supramolecular side chain to side chain interactions.[193–197] Recently, an approach called genetically encoded protein stapling[198] was introduced. In this technique a non-natural electrophilic amino acid is incorporated into a protein sequence to enable a ligation reaction with a nucleophilic residue in proximity (e.g. lysine, histidine, or cysteine). The resulting intramolecular cross-link was designed to stabilize an α-helical stretch within a protein domain. In general, both the location and linker length of the bridges between the side chains have to be chosen carefully to avoid interference with target binding and to facilitate efficient helix stabilization. Approaches that have been successfully applied to the generation of PPI inhibitors involve thiol-, lactam-, as well as triazole-based cross-links and hydrocarbon staples.

##### 2.3.1.1. Thiol-Based Cross-Links

**Figure 8:**
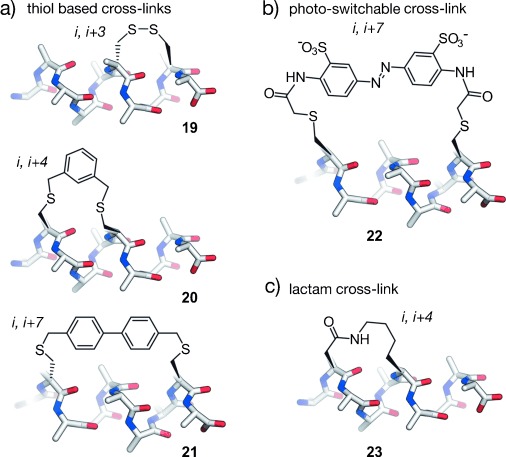
Thiol- and lactam-based cross-linked α-helical peptides: a) Disulfide linkage of d- and l-cysteine at positions *i*, *i*+3 (19), *m*-xylene-cross-linked l-cysteines at positions *i*, *i*+4 (20), diaryl cross-linked d-cysteine and l-cysteine at positions *i*, *i*+7 (21). b) Azobenzene-based cross-linked l-cysteines at positions *i*, *i*+7 (22). c) Lactam formed between aspartic acid and lysine at positions *i,* *i*+4 (23).

One of the first conformationally constrained helical peptides was generated by disulfide formation between 2-amino-6-mercaptohexanoic acid introduced at position *i* and cysteine at position *i*+7. To ensure the correct alignment for cross-linking, 2-amino-6-mercaptohexanoic acid was used as the d-amino acid. Cross-linked peptides show an increased α-helical content compared to their acyclic counterparts.[191] The cross-linking of d- and l-cysteine at positions *i* and *i*+3, respectively, also proved useful for the conformational stabilization of α-helical peptides (**19**; Figure 8 a).[199] The linker length can influence helicity and target recognition. Longer disulfide cross-links were obtained by replacing cysteine with homocysteine.[200] Disulfide cross-links are labile under the reductive conditions found in the cytosol of most eukaryotic cells. For this reason, chemically more stable thioether moieties were used to replace the disulfides. Again, cysteine can be replaced by homocysteine, to maintain the linker length of the disulfide bridge.[201] Cysteine possesses a unique reactivity among the proteinogenic amino acids, which allows the design of electrophiles that selectively react with the thiol side chain. A variety of biselectrophilic molecules were used to cross-link two properly aligned cysteines, with the aim of stabilizing the helical conformation. Biselectrophilic mono- and diaryl linkers have been used to provide class A peptidomimetics with increased α-helical character. The highest α-helicity for cross-linked peptides bearing l-cysteines located at positions *i* and *i*+4 is observed for structurally rigid linkers such as *m*-xylene, as determined by circular dichroism (CD) and NMR spectroscopy (**20**; Figure 8 a).[202] Longer cross-links, such as from bisarylmethylene bromides, gave the best results for peptides with d-cysteine at position *i* and l-cysteine at *i*+7 (**21**; Figure 8 a). These cross-linked peptides show increased cellular uptake and potential as PPI inhibitors.[203–205] Perfluorinated aryl linkers are also used to stabilize α-helices, thereby conferring increased protease resistance and cellular uptake.[206] The switchable azobenzene scaffold represents a special case of thiol-based cross-links (**22**; Figure 8 b).[207–213] When used as a cross-link, photoisomerization between the *cis* and *trans* isomers of the azobenzene moiety holds the potential to modulate the helical character of a peptide. The azobenzene cross-link has been introduced as bis(iodoacetamide) or bis(chloroacetamide), which reacted with cysteines at positions *i* and *i*+7 or *i* and *i*+11. In the *i*, *i*+7 setup, the α-helical conformation was shown to be more pronounced with the *cis*-azobenzene moiety, whereas the *trans* configuration led to increased α-helicity for *i*, *i*+11 cross-linking.[214] These azobenzene-cross-linked peptides proved useful as switchable PPI inhibitors.[214]

##### 2.3.1.2. Lactam Cross-Links

Contemporaneously to disulfide-bridged peptides, the formation of an amide bond between lysine and aspartic acid at positions *i* and *i*+4, respectively, was described to induce α-helicity (**23**; Figure 8 c).[190] Another architecture involves the reaction of two glutamic acid residues at positions *i* and *i*+7 with a diamino-functionalized building bock forming two amide bonds within one cross-link.[215, 216] Potent PPI inhibitors have been obtained by the introduction of two adjacent lactam cross-links.[217] A similar approach introduces the two lactam cross-links in an overlapping fashion, thereby generating highly helical peptides.[218]

##### 2.3.1.3. Triazole Cross-Links

[1,2,3]-Triazoles formed by means of copper-catalyzed azide–alkyne [3+2] cycloaddition, also known as click chemistry, are valuable structures in organic synthesis and drug discovery[219, 220] that have also been used for the stabilization of α-helices. It was shown that replacement of lactam cross-links by [1,2,3]-triazole rings provides peptides with similar α-helical content.[221] For further increased α-helicity, two triazole cross-links were introduced in a single peptide, thereby resulting in more affine binders with enhanced protease stability.[222] Furthermore, peptides containing two azido groups at positions *i* and *i*+7 can react with linkers bearing two alkyne moieties in a “double-click” reaction. Notably, this reaction allows the versatile introduction of additionally functionalized linkers.[223, 224] Cell penetration of the modified peptides was achieved by attaching arginines to an aromatic linker to yield active peptides, as shown by a reporter gene assay.[225, 226]

##### 2.3.1.4. α-Methylated Hydrocarbon Cross-Links

**Figure 9:**
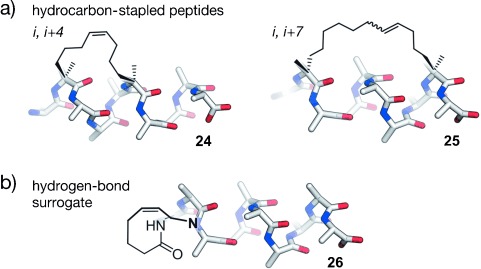
RCM cross-linked α-helical peptides: a) Hydrocarbon-stapled peptides: Cross-linked α-methylated building blocks at positions *i*, *i*+4 (24) and *i*, *i*+7 (25); b) hydrogen-bond surrogate: Covalent replacement of the hydrogen bond between the N-terminal amino acid (*i*) and the amine proton at position *i*+3 (26).

Verdine and co-workers introduced the so-called hydrocarbon peptide stapling technique (Figure 9 a).[192] This approach combines two features for the stabilization of α-helices: 1) The methylation of α-carbon atoms and 2) the introduction of a covalent side chain to side chain cross-link. The synthesis of stapled peptides involves the incorporation of α-methyl-α-alkenylamino acids during solid-phase peptide synthesis. In analogy to the previously reported ring-closing olefin metathesis (RCM) of homoserine *O*-allyl ethers,[227, 228] these modified amino acids are cross-linked by RCM. It was observed that a minimal cross-link length is required for high RCM conversion and that not all macrocyclic peptides show increased α-helicity. The best results were achieved with architectures that involve modifications at positions *i* and *i*+4 or *i* and *i*+7. For stapled peptides with cross-links spanning one turn of a helix, two *S*-configured non-natural amino acids are incorporated at positions *i* and *i*+4 by employing a C_8_ linker (**24**). A bridging of two turns of a helix is achieved by incorporating an *R*-configured building block at position *i* and an *S*-configured one at *i*+7 (**25**). This architecture requires the use of C_11_ linkers.[192] The use of *i*, *i*+3 stapled peptides has also been described with an *R*-configured amino acid at position *i* and an *S*-configured one at *i*+3 cross-linked by C_8_ or C_6_ linkers.[229, 230] Recently, *i*, *i*+3 stapled peptides were also used as PPI inhibitiors.[87, 231] An expansion of the stapling technology uses either two isolated hydrocarbon cross-links simultaneously[232] or two cross-links that are connected through a central spiro ring junction to generate so-called stitched peptides.[233] Compared to conventionally cross-linked peptides, stitched peptides show further increased chemical and proteolytic stability as well as increased cellular uptake. However, this approach has not yet been used for the development of PPI inhibitors. Hydrocarbon-stapled peptides show enhanced α-helicity, improved protease stability, and, in many cases, increased cellular uptake compared to their natural precursors.[234–237] However, in some cases, extensive sequence optimization is required to ensure efficient cell permeability.[234, 238] As a consequence of the robustness of the RCM reaction[239] and improved pharmacokinetic properties of hydrocarbon-stapled peptides, the technique was frequently applied for the stabilization of α-helical peptides and has proven particularly useful for the development of PPI inhibitors.

#### 2.3.2. N-Terminal Caps

The analysis of structural data revealed that protein residues with hydrogen-bonding capabilities show a high propensity to occur at the N-terminal end of an α-helix. This can be explained by side chain to backbone hydrogen bonds that nucleate helix formation.[85, 86, 240] For example, aspartic acid or asparagine at the most N-terminal position (*i*) within an α-helix often show hydrogen bonding to the backbone amide at position *i*+2. The introduction of artificial N-terminal capping motifs, so call N caps, was used to nucleate α-helixes. These N caps are capable of stabilizing several turns of an α-helix since helix nucleation is the energetically most demanding step.[241] The most successfully used N caps for the generation of PPI inhibitors are hydrogen-bond surrogates (HBS), in which the hydrogen bond between the N-terminal amino acid (*i*) and the amine proton at position *i*+3 is replaced by a covalent linker. Various linker types have been applied as surrogates. Of these, hydrazones and thioethers with hydrocarbon cross-links formed by means of RCM (**26**; Figure 9 b) represent the most stabilizing scaffold.[241–243] The latter approach provides peptides with increased target affinity and bioavailability and allows a stabilization of the α-helical conformation without sacrificing side chains.[244–246] This is particularly interesting for helical peptides that have most of their residues involved in target recognition.[247]

#### 2.3.3. Foldamers

**Figure 10:**
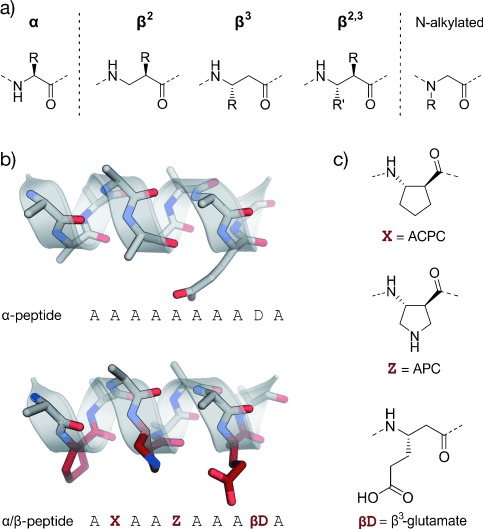
Foldamers: a) Amino acids used in α- and β-peptides as well as peptoids (N-alkylated); b) α- and α/β-peptide in stick representation (schematic representation of the helix is shown transparent). β-Amino acids are highlighted in red; c) β-amino acids commonly used in α/β-peptides (ACPC: *trans*-2-aminocyclopentanecarboxylic acid, APC: *trans*-3-aminopyrrolidine-4-carboxylic acid, βD: β^3^-glutamate as an example of β^3^-amino acids).

Foldamers are defined as non-natural oligomeric structures with predictable folding propensities.[248–252] Among foldamers, β-peptides, peptoids, hybrids such as α/β-peptides, and mixtures of α-peptides and peptoids proved suitable scaffolds for the development of inhibitors of helix-mediated PPIs. These non-natural oligomers combine the folding properties of α-helical peptides with valuable features such as proteolytic[253] and metabolic resistance,[254] which may be challenging to achieve with their natural analogues (Figure 10).

##### 2.3.3.1. β-Peptides

β-Peptides are the most exhaustively studied foldamers with well-characterized folding propensities.[255, 256] They are synthesized by the consecutive coupling of β-amino acids bearing an additional backbone methylene group compared to α-amino acids. Different types of β-amino acids have been described: β^2^- or β^3^-building blocks bearing a single side chain either at C_2_ or C_3_, and β^2, 3^-amino acids with both carbon atoms being substituted. β-Peptides can fold into several helical conformations such as the 14-helix (3_14_-helix) or the 12-helix (2.5_12_-helix). 14-Helices consisting of β^3^-amino acids are stabilized by 14-membered rings formed through hydrogen bonds between the amide proton at position *i* and the carbonyl oxygen atom at position *i*+2, thereby including three residues per turn. As α-helices, this 14-helix exhibits left-handed chirality but an opposite net macrodipole. It orients the side chains into three faces of the helix, thus allowing their interaction when placed on the same face of the helix. The 12-helix is stabilized by hydrogen bonds between the carbonyl oxygen atom at position *i* and the amide proton at position *i*+3, thereby resulting in 2.5 residues per turn. The orientation of the macrodipole is similar to an α-helix. In organic solvents, β-peptides exhibit a more pronounced tendency to fold into helices than their α-peptide counterparts. However, the folding tendency of β-peptides in aqueous solutions appears to be lower than that of α-peptides. Thus, different approaches have been developed to promote the proper folding of β-peptides in aqueous solutions: Introduction of cyclic β-amino acids,[248, 257] incorporation of intramolecular salt bridges (β^3^-Glu/β^3^-Lys and β^3^-Glu/β^3^-Orn),[258–260] placement of a γ-branched β^3^-amino acids at the first side chain carbon atom, assembly of hydrocarbon or diether cross-links,[261, 262] and stabilization of the helix macrodipole.[263]

Conformational control and the preference for a certain type of helix can be achieved by the introduction of cyclic β-amino acids with different steric demands. Thus, the six-membered ACHC residue favors the 14-helix formation, while the five-membered ACPC favors the 12-helix (ACHC=*trans*-2-aminocyclohexanecarboxylic acid. Similar to α-peptides, one of the main disadvantages of β-peptides is their poor cell permeability. The introduction of cationic patches and hydrocarbon or diether bridges can improve cellular uptake.[262]

##### 2.3.3.2. α/β-Peptides

The combination of α- and β-amino acids in one oligomer generates a wide range of heterogeneous foldamers with different and predictable folding properties which can be easily modulated by altering their design.[264–266] Thus, α/β-peptides were developed to improve the mimicry of an α-helix while ensuring enhanced resistance to proteolysis.[267] α-Amino acids are used for surface recognition, while the β-amino acids (mainly rigid cyclic β-amino acids and β^3^-Glu-β^3^-Lys pairs to form intramolecular salt bridges) are incorporated to support the helical conformation.[268] These scaffolds require fewer residues than α-peptides to fold into helices.[269] Notably, building blocks such as acyclic β^3^-amino acids and β-branched α-amino acids decrease helicity, while α,α-disubstituted residues enhance the folding properties.[270] Combinations of α/β- with α-peptides, and patterns such as “ααβαααβ” or “αααβ” also proved useful for the design of various PPI inhibitors.

##### 2.3.3.3. Peptoids

Peptoids are composed of α-amino acids which bear their side chains at the amide nitrogen instead of the α-carbon atom.[271–273] These foldamers allow a high degree of diversification, are highly resistant to proteolysis, and exhibit improved cell permeability. Peptoids also fold into chiral helices[274] similar to the type I polyproline helix. The amide bonds adopt a *cis* geometry and the macrodipole is oriented opposite to the α-helical peptides. The presence of chiral side chains supports the helical conformation independent of hydrogen-bond patterns, thus making it persistent in a variety of solvents and at a broad pH range. Mixtures of α-amino acids and peptoid monomers also proved to be potent PPI inhibitors.[275, 276]

#### 2.3.4. Structural Helix Mimetics

**Figure 11:**
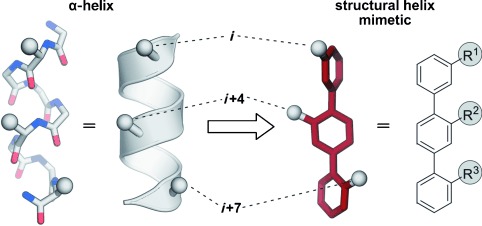
Concept of structural α-helix mimetics (class C): Left: Stick and schematic representations of an α-helix. The side chains *i*, *i*+4, and *i*+7 are represented by a sphere. Right: Stick representation and chemical structure of a terphenyl structural mimetic. The substituents R^1^, R^2^, and R^3^ mimic the tridimensional projection of side chains *i*, *i*+4, and *i*+7 of an α-helix.

While foldamers still have peptidic character, Hamilton and co-workers suggested a completely different scaffold for mimicking an α-helix. They reported the replacement of the entire peptide backbone by a rodlike structure with small-molecule character that mimics the side chain projection of residues with a relative spacing of *i*, *i*+4 (or *i*+3), and *i*+7 (Figure 11).[277] The goal was the design of mimetics that accurately reproduce the surface of an α-helix, and benefit from modular as well as divergent syntheses and improved pharmacokinetic properties (e.g. proteolytic resistance and oral availability). The first mimetic was based on a functionalized terphenyl (Figure 11, right). Later, more scaffolds were tested for their ability to mimic an α-helix. In accordance to the major driving force responsible for conformational rigidity, these structures are classified in the following groups: sterically enforced, hydrogen-bond guided, and covalently constrained scaffolds. In some scaffolds, a combination of these driving forces may occur. Again, we will focus our attention on the structures that proved useful for the development of PPI inhibitors. For a global overview of structural mimetics of α-helices, see the corresponding reviews.[278, 279]

##### 2.3.4.1. Sterically Enforced Scaffolds

**Figure 12:**
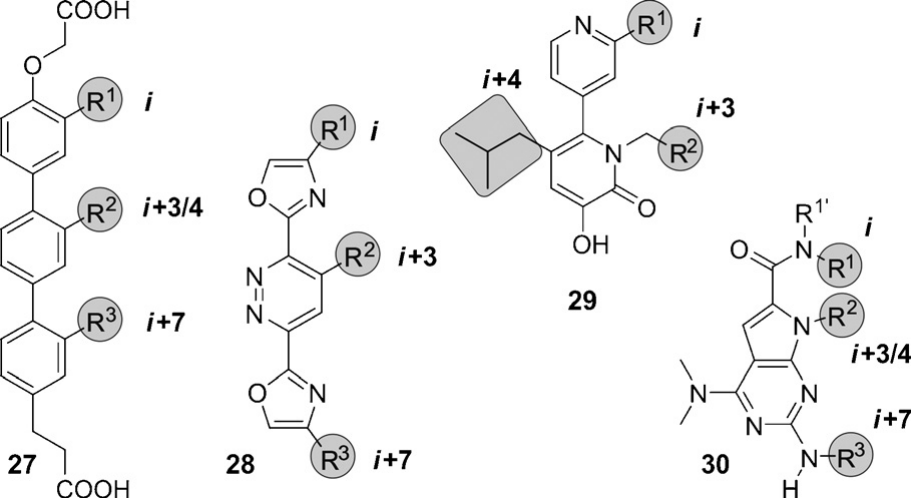
Chemical structures of sterically enforced structural α-helix mimetics: A terphenyl (27), two different heterocyclic scaffolds (28 and 29), and a pyrrolopyrimidine (30). The substituents highlighted in gray are designed to mimic the *i*, *i*+3/4, and *i*+7 side chains of an α-helix.

In terphenyl scaffold **27**, the conjugation of the aromatic rings promotes coplanarity of the phenyl rings, while the steric interactions between *ortho* substituents favor its nonplanarity (Figure 12).[277] A major contribution of the second effect results in a staggered conformation that suitably mimics two turns of an α-helix. Highly potent terphenyl-based inhibitors have been developed for a number of PPIs. However, these low-molecular-weight structures exhibit a high degree of conformational heterogeneity and require long and tedious synthetic routes involving the formation of many C–C bonds.[280, 281] In addition, terphenyls are highly hydrophobic and poorly water soluble. Thus, the development of alternative structural mimetics of α-helices was pursued. In an effort to improve the water solubility and to reduce the synthetic complexity, the aromatic rings in terphenyls were replaced by five- or six-membered heterocycles (Figure 12). These amphiphilic heterocyclic scaffolds (**28**) keep one side of the molecule as the interacting surface and accumulate a number of heteroatoms on the opposite side, thereby increasing the polarity.[282–285] In analogy to terphenyls, steric factors represent the major contribution for the spatial preorganization of these scaffolds. Additionally, new α-helix side chain patterns have been implemented. For example, Becerril and Hamilton designed a series of pyridylpyridone-derived compounds mimicking the *i*, *i*+3, and *i*+4 positions of an α-helix (**29**). X-ray crystallography confirmed suitable mimicry of the bioactive conformation of the LXXLL motif originating from coactivator proteins of the estrogen receptor (ER) with a root-mean-square (rms) deviation of 0.36 Å.[286] These scaffolds have also been used to target hydrophilic PPIs for the first time.[287] Later, Lim and co-workers designed pyrrolopyrimidines (**30**),[288] which has a more rigid scaffold with an hetero-bicyclic structure that mimics the orientation of the residues *i*, *i*+3 (or *i*+4), and *i*+7 of an α-helix (Figure 12). A straightforward solid-phase synthesis that facilitates the generation of libraries has also been described. Notably, these water-soluble compounds have been proven to be cell-permeable.

##### 2.3.4.2. Hydrogen-Bond-Guided Scaffolds

**Figure 13:**
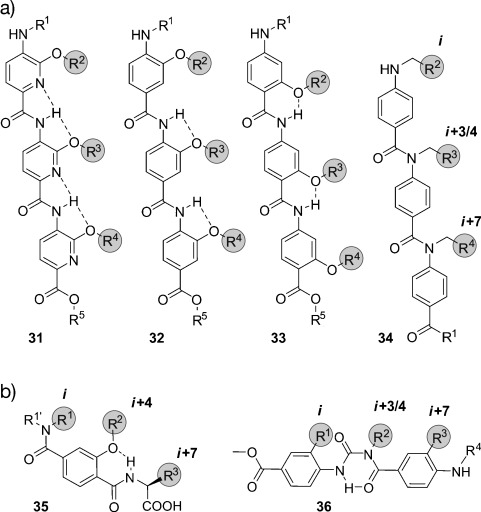
Chemical structures of hydrogen-bond-guided structural α-helix mimetics: An oligopicolinamide or trispyridylamide (31), a 3-O-alkylated oligobenzamide (32), a 2-O-alkylated oligobenzamide (33), an N-alkylated oligobenzamide (34), a terephthalamide (35), and a benzoylurea (36). The substituents highlighted in gray are designed to mimic the *i*, *i*+3/4, and *i*+7 side chains of an α-helix.

Oligoamides (**31**–**34**) were designed as straightforward synthetically accessible scaffolds (Figure 13 a) that allow the employment of solid-phase synthesis, thereby promoting diversification of side chains and access to compound libraries.[289] Intramolecular hydrogen bonds ensure the presentation of substituents on the same face of the molecule, thus enabling α-helix mimicry. There are four main subclasses of oligoamides: Oligopicolinamides or trispyridylamides (**31**),[289] 3-O-alkylated oligobenzamides (**32**),[290–292] 2-O-alkylated oligobenzamides (**33**),[293] and N-alkylated oligobenzamides (**34**).[294, 295] Structural investigations confirm that the trispyridylamide scaffold (**31**) is rigidified by two hydrogen bonds centered around the amidic proton (Figure 13). Interestingly, these hydrogen bonds induce a severe structural constraint that results in a pronounced curvature of this scaffold and the eclipsed disposition of the substitutions that would mimic the *i*, *i*+4 (or *i*+3), and *i*+7 positions of an α-helix. As a result of the presence of only one hydrogen bond, the O-alkylated oligobenzamides (**32** and **33**) are more flexible and exhibit reduced curvature. The combination of pyridine and phenyl rings in one scaffold allows the adjustment of the backbone curvature,[296] which can also be achieved by changing the substitution pattern of the aromatic rings. Thus, 2-O-alkylated oligobenzamides (**33**) have a lower backbone curvature than the 3-O-alkylated oligobenzamides (**32**). Compared to the initial trispyridylamides, a mixture of pyridine and phenyl rings increase the hydrophobicity and the flexibility of the scaffold, which allows a more staggered disposition of the substituents and leads to more potent PPI inhibitors. N-Alkylated oligoamides (**34**) are the structurally simplest oligoamides described so far. Attempts to functionalize the non-recognition face of these scaffolds have been described by Wilson and co-workers.[297, 298] However, the improvement in solubility is associated with a concomitant decrease in the inhibitory activity. A dimeric mimetic of N-alkylated oligoamides lacking positive cooperativity has also been synthesized by means of click chemistry.[299] Wilson and co-workers recently reported a hybrid mimetic that combines monomers from 2-O-alkylated, 3-*O*-alkylated, and N-alkylated oligoamides and α-amino acids, thereby highlighting the significance of stereogenic substituents.[300] Additional oligoamide-based scaffolds have been described as PPI inhibitors. Thus, DeGrado and co-workers suggested a thioester-substituted arylamide,[301] Hamilton′s group has also reported a biphenyl-4,4′-dicarboxamide scaffold,[302] while Whitby and Boger proposed an extremely simplified version of a 3-O-alkylated oligobenzamide[303] that has enabled straightforward access to large libraries of compounds for screening PPI inhibitors. In summary, oligoamides have been shown to be less potent PPI inhibitors than the terphenyl scaffolds. However, their synthetic simplicity and the straightforward diversification of the substituents represent valuable properties that increase the applicability of these scaffolds.

Terephthalamides (**35**; Figure 13 b) are helix mimetics with increased rigidity as well as aqueous solubility, and with reduced synthetic complexity compared to the terphenyl scaffold.[304] The restricted rotational freedom around the amide bonds and the intramolecular hydrogen bond preorganize the scaffold such that the functionalities reproduce the spatial orientation of the *i*, *i*+4, and *i*+7 residues of an α-helix. Potent inhibitors of PPIs have been obtained with terephthalamides, comparable to the ones based on the terphenyl scaffold, but with improved solubility and improved synthetic accessibility. In α-helix-mediated PPIs, critical interactions are established by a number of residues, generally exceeding the three side chains that are typically mimicked by nonpeptidic PPI inhibitors (*i*, *i*+4, and *i*+7). Thus, benzoylureas (**36**) are attractive mimetics that allow facile and consecutive elongation of the scaffold (Figure 13 b) to yield water-soluble structures with highly asymmetric substitution (if desired).[305] The aromatic rings of the terphenyls are replaced by a six-membered hydrogen-bond-assisted ring that provides some degree of rigidity and ensures the staggered disposition of the substituents. In addition, the hydrophilicity is increased. Modular synthesis to access amphiphilic benzoylureas simultaneously mimicking the *i*, *i*+1, *i*+4, *i*+6, and *i*+8 or the *i*, *i*+1, *i*+4, *i*+7, and *i*+8 positions of an α-helix have been described.[306] Benzoylureas are highly valuable scaffolds as they represent the most complete mimicry of two α-helical turns described so far. However, the development of PPI inhibitors based on these benzoylureas has not yet been described.

##### 2.3.4.3. Covalently Constrained Scaffolds

**Figure 14:**
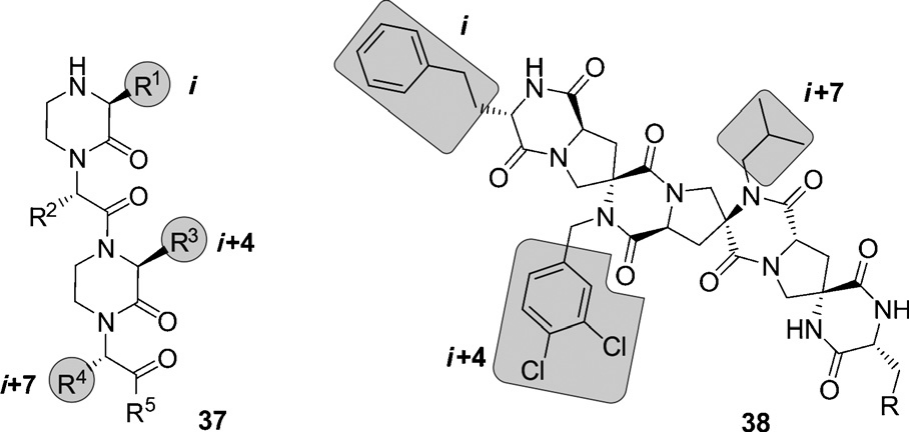
Chemical structures of covalently constrained structural α-helix mimetics: An oligooxopiperazine (37) and a spiroligomer (38). The substituents highlighted in gray are designed to mimic the *i*, *i*+3/4, and *i*+7 side chains of an α-helix.

Oligooxopiperazines (OHMs, **37**) were first reported by Tošovská and Arora (Figure 14).[307] The absence of aromatic rings in their structure and a chiral backbone are two prominent features of this amino acid derived scaffold. Structural studies confirmed the mimicry of residues *i*, *i*+4, and *i*+7 of an α-helix when the amides adopt the preferred *trans* geometry. An oxopiperazine dimer has a similar length as an octameric α-helix and exhibits a well-defined structural arrangement that is strongly assisted by the cyclization of the peptide backbone. The chirality of the structure may confer higher binding specificity and represents a novel and valuable feature of this scaffold. Spiroligomers (**38**) represent another example of a covalently constrained chiral scaffold (Figure 14).[308] The constituent spiro monomers determine the three-dimensional structure, thereby fixing the orientation of substituents. In the so-called “assembly stage”, bis(amino acid)s are consecutively coupled on a solid support. The “rigidification stage” subsequently closes the diketopiperazine rings to obtain the final rigid scaffold.[309, 310] A new reaction compatible with solid-phase synthesis has been developed for highly hindered diketopiperazines (with five or six substituents).[310] Remarkably, confocal microscopy indicates a good cell permeability of the scaffold through passive diffusion.[311] Finally, the chirality of the structure has a significant effect on their activities, thus reinforcing the assumption that chiral scaffolds may have higher inhibitory potential. In addition, Zhang and co-workers have described a structural mimetic based on cross-acridine, which was functionalized at the termini and mimics the *i*, *i*+3, *i*+5, and *i*+7 positions of an α-helix. The structure is achiral and aromatic, but the mimicry is assisted by the presence of double or amide bonds with restricted rotational freedom and by the strong rigidity of the multicyclic scaffold.[312]

## 3. Targeted Protein–Protein Interactions

Peptide-inspired PPI inhibitors were developed for a broad range of targets. Some proteins, such as G-protein-coupled receptors (GPCR), apoptosis regulators MDM2/MDMX, and BCL-2 family proteins, evolved into model systems that were widely used to test the applicability of peptidomimetics. Here we will discuss the development of class A–C mimetics for these model systems and additional targets involving small GTPases, transcriptional regulators, enzymes, and pathogenic proteins. This section does not present all the examples of peptide-derived PPI inhibitors comprehensively, but focuses on major target classes and recent contributions.

### 3.1. Transmembrane Receptors

Transmembrane receptors are involved in important signaling processes that connect extracellular events with intracellular responses. Their malfunction is implicated with numerous pathogenic states that range from metabolic disorders to cancer.[313–316] Receptors respond to the binding of effectors such as small molecules, peptide hormones, or protein ligands. In some cases, the activation of receptors requires the binding of additional cofactors, thus adding complexity to the signaling networks. Several PPI inhibitors derived from peptide sequences that are recognized by receptors have been described. Examples include helical β-peptides that inhibit the interaction between the scavenger receptor B and high-density lipoprotein,[317] or α/β-peptides that target the receptor binding site of vascular endothelial growth factor.[90] A hyperactivity of epidermal growth factor receptor (EGFR) tyrosine kinase is implicated in the onset and progression of numerous types of cancer.[314] EGFR inhibitors usually target the extracellular receptor binding site,[318] the intracellular adenosine triphosphate binding site,[314, 319] or the interaction between EGFR and cofactor Grb2 (growth factor receptor bound protein 2).[320] A crucial step for the receptor activity is the dimerization mediated by a coiled coil structure.[321] Schepartz and co-workers introduced all-hydrocarbon-stapled peptides capable of inhibiting this dimerization.[322] In addition, these peptides proved active in cell-based assays.[323] Interestingly, the corresponding peptide with an open cross-link bearing the two olefin side chains showed similar activity in these assays.[324] Recently, a nonhelical, triazolyl-bridged peptide was developed, also targeting EGFR dimerization.[157]

GPCRs resemble a large family of transmembrane receptors that are activated by a multitude of different ligands, also including peptide hormones. A variety of inhibitors of peptide ligand/receptor interactions have been developed, which are beyond the scope of this Review and have been extensively reviewed by Fairlie and co-workers.[70] Notably, the similarity in the interactions of receptors with peptides and proteins suggests that the concepts for interfering with peptide–receptor interactions may also be applicable for the design of PPI inhibitors. One of these examples involves the incorporation of benzodiazepines into Angiotensin II, since it was known that the bioactive conformation of Angiotensin II contains a β-turn. The final peptidomimetic showed affinity for AT1 and AT2 receptors.[325] Another example involves the use of a glucose scaffold presenting Somatostatin side chains in a β-turn conformation, which results in an agonist of the Somatostatin receptor.[77] A library based on the *trans*-pyrollidine-3,4-dicarboxamide scaffold led to high-affinity ligands for human opioid receptors.[83] Both the glucose and the *trans*-pyrollidine-3,4-dicarboxamide are structural-turn mimetics (class C).[83] GPCR protein effectors such as the melanocortin receptor (MCR) are also known to interact with agouti (ASP) and agouti-related protein (AGRP). The NMR structure of the C-terminal binding site reveals a cysteine knot presenting three crucial residues in a turn structure.[326, 327] The isolated binding motif can be chemically stabilized by a substitution of the disulfide by a lactam bridge.[328] Other GPCRs recognize binding partners through their helical interaction domains. A hydrocarbon-stapled peptide with enhanced agonist potency was discovered in the case of the agonists of vasoactive intestinal peptide receptor 2 (VPAC_2_),[329] whereas helical α/β-peptides were able to inhibit the interaction between parathyroid hormone and the parathyroid hormone-related peptide receptor.[330]

**Figure 15:**
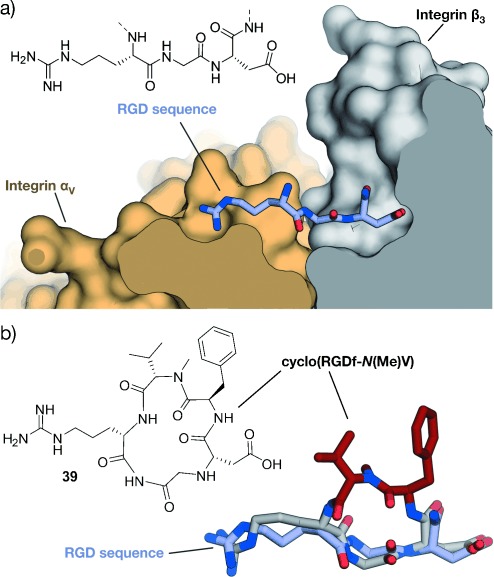
RGD–integrin interaction: a) Crystal structure of the RGD sequence from fibronectin bound to the α_V_ (orange) and β_3_-subunit (gray) of the integrin receptor (PDB 4MMX). b) Chemical structure of the cyclic pentapeptide cyclo(RGDf-N(Me)V) and crystal structures (gray/red, PDB 1L5G)[341, 342] superimposed with fibronectin RGD (gray; red=constraining amino acids; f=d-phenylalanine).

Integrins play an important role in the interaction of extracellular matrix protein with the cell surface and in cell–cell adhesion in vertebrates. Misregulation of certain integrin receptors is linked to several diseases, including cancer.[313] Integrins are composed of an α- and a β-subunit and many of them recognize binding partners through an Arg-Gly-Asp (RGD) sequence (Figure 15 a).[331] To install conformational constraints, Kessler and co-workers integrated the RGD sequence into cyclic pentapeptides, thereby increasing the activity and bioavailability.[52, 332] Further optimization efforts that were assisted by NMR-based structural investigations resulted in the identification of the macrocyclic inhibitor cyclo(RGDfV) called Cilengitide.[333] Notably, the d-phenylalanine (f) is involved in additional hydrophobic contacts with the target and it contributes to the conformational rigidity of the macrocycle. The valine side chain is not involved in direct interactions,[334] which allows its substitution by lysine and thus enables the attachment of labels.[335] Other modifications such as the replacement of amide bonds by thioamides, retroinversion,[336] or the introduction of turn-inducing amino acids or turn mimetics[334, 337] lead to a changed conformation, thereby resulting in reduced target affinity. Finally, N-methylation of the valine, to give the cyclic pentapeptide cyclo(RGDf-*N*(Me)V), combines high receptor affinity and selectivity with improved biostability and oral availability (Figure 15 b).[67] An alternative approach to constrain the RGD sequence is its incorporation into the so-called “cysteine ladder” peptides.[46] These naturally occurring cyclic peptides compose several disulfide bridges arranged in a parallel fashion. In addition to the RGD sequence, integrin receptors can recognize the LDV turn structure.[338] Based on this minimal sequence, cyclic peptides were developed containing BTD (β-turn dipeptide) as the turn-inducing element.[339] Others include a cyclization of the three amino acid turn backbone, with the side chain orientation maintained.[340]

### 3.2. Apoptosis Regulation

#### 3.2.1. MDM2 and MDMX

**Figure 16:**
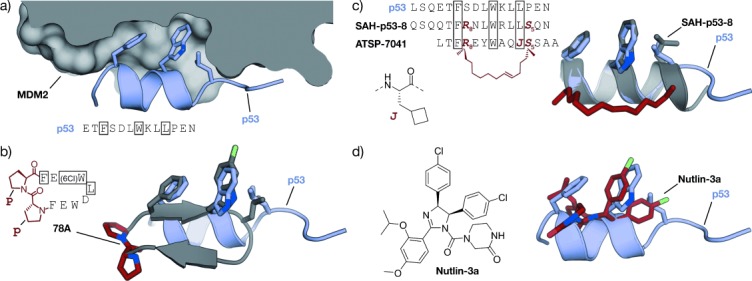
MDM2–p53 interaction: a) Crystal structures of MDM2 (gray) with the transactivation domain of p53 (blue, PDB 1YCR).[347] b) Superimposed crystal structures of p53 (blue, PDB 1YCR) and cyclic β-hairpin peptide 78A (gray/red, PDB 2AXI). The d-Pro-l-Pro (p-P) cross-link is highlighted in red.[115] c) Sequences of stapled peptides (left). Superimposed crystal structures (right) of p53 (blue, PDB 1YCR) and SAH-p53-8 (gray/red, PDB 3V3B). The cross-link is highlighted in red (side chains of amino acids in boxes are shown explicitly in the crystal structures).[355] d) Superimposed crystal structures of p53 (blue, PDB 1YCR) and Nutlin-3a (red, PDB 4HG7).[356] All superimposed structures were obtained from structures of complexes with MDM2 or MDMX.

MDM2 and MDMX (also known as MDM4 and HDM4/HDMX) downregulate the tumor suppressor p53. In response to cellular stress, the transcription factor p53 mediates the expression of genes involved in protective processes such as DNA repair, cell cycle arrest, and apoptosis.[343, 344] Binding of MDM2 and MDMX to the N-terminal transactivation domain of p53 blocks this so-called “guardian of the genome”, either by mediating its ubiquitylation that finally leads to its degradation by the proteasome[345] or by acting as a direct antagonist.[346] An upregulation of MDM2 and MDMX has been detected in many types of cancers, thus resulting in the interaction between these proteins and p53 being prime targets for anticancer strategies. Crystal structures of the complex between MDM2 and the transactivation domain of p53 reveal an α-helical conformation of the p53 interaction domain when bound to MDM2 (Figure 16 a).[347] P53 hot-spot residues involve Phe19, Trp23, and Leu26.[347] This structural information together with the crystallographic data of the similarly arranged p53–MDMX complex[348] have been used as the starting point for a rational design of the corresponding PPI inhibitors. For some peptidomimetics, helical peptides derived from phage-display selections served as alternative starting points. Examples include the phage-display-derived peptides pDi[349] and PMI[350] that exhibit dual inhibitory effects for both the p53–MDM2 and p53–MDMX complexes. This is considered a desirable feature for efficient anticancer activity. Additionally, mirror-image phage-display (MIPD) techniques together with native chemical ligation have provided proteolytically more-resistant d-peptide inhibitors of the p53–MDM2 interaction. However, these peptides do not feature sufficient cell permeability.[351–353] Finally, although mRNA display has enabled the screening of larger libraries of peptides,[354] the proteolytic instability and/or poor cellular uptake of these peptides remain major limitations of these approaches.

A variety of peptidomimetics were designed based on these peptide binders. An early example of a class A mimetic involves a modified octapeptide comprising four non-natural amino acids that binds HDM2 in vitro with nanomolar affinities.[357–359] This peptide mediates the accumulation of p53 in cancer cells, thereby promoting cell death by apoptosis.[360] Again, low cellular uptake and low proteolytic resistance are the main drawbacks of this peptide. Later, Robinson and co-workers were able to graft the crucial residues of the p53 helix onto a cyclic β-hairpin. Head-to-tail macrocyclization and the d-Pro-l-Pro (p-P) turn mimetic were employed to stabilize the β-sheet structure, which displays good affinity and binds HDM2 at the p53 binding site.[114] Sequence optimization by the introduction of non-natural amino acids yielded class B mimetics with improved affinities (Figure 16 b).[115] Remarkably, this innovative approach represents one of the few examples of a stabilized β-sheet structure used as a PPI inhibitor, and it impressively illustrates the interchangeability of secondary structures.

The use of thiol- and triazole-based cross-links and the peptide-stapling technique for the generation of class A helix mimetics were evaluated to overcome the limitations regarding cellular permeability and proteolytic resistance. The incorporation of a bisaryl cross-link at positions *i* and *i*+7 of the pDi sequence resulted in peptides with only a modest enhancement of α-helicity and bioactivity, but a prominent increase of cellular uptake. A d,l-dicysteine-linked 6,6′-bis(bromomethyl)-3 3′-bipyridine (Bpy) cross-link contributes with additional contacts to MDMX, thereby yielding more affine binders.[203] A cross-linking based on photoinduced 1,3-dipolar cycloaddition provides peptides with high affinities for MDM2 and MDMX that displayed improved cellular uptake and dual inhibitory activity in cells after the incorporation of positively charged amino acids.[361] A double triazole tethering approach enables the synthesis of several cross-linked peptides based on a single p53-derived sequence by using a set of modified linkers. The resulting cross-linked peptides exhibit affinities comparable to the wild-type peptide combined with improved proteolytic stability. Furthermore, the incorporation of Arg moieties in the linker resulted in cell-penetrating peptides, thus omitting the need for additional sequence variations.[225, 226] Alternatively, HBS-stabilized helices[362] and metallopeptides[194] also led to binders of MDM2.

A series of stapled α-helical p53-derived peptides (SAH-p53, Figure 16 c) with cross-linking positions *i* and *i*+7 showed increased α-helicity, improved binding affinity for MDM2, and enhanced proteolytic stability when compared with the wild-type p53 peptide. After replacement of negatively charged amino acids, the resulting neutral or positively charged stapled peptides feature cell permeability, induce apoptosis, and suppresses tumor growth in vivo.[363, 364] Remarkably, crystallographic data revealed a direct involvement of the hydrocarbon cross-link in MDM2 binding, thus explaining the dramatic increase in affinity upon incorporation of a staple.[355] Another set of stapled peptides with profound alterations in the side chains serve as dual inhibitors of MDM2 and MDMX.[365] Recently, Aileron Therapeutics reported the development of another series of stapled peptides based on phage-display-derived peptide pDi. For some candidates, for example, ATSP-7041, they describe high specificity and affinity for both MDMX and MDM2 and improved pharmacokinetic properties. One candidate of this series is currently being tested in clinical trials.[365] Moreover, ATSP-peptides bind to mutated forms of MDM2 that are not accessible for small-molecule p53–MDM2 inhibitors of the Nutlin family (Figure 16 c).[238, 366] Nutlins are class D peptidomimetics capable of inhibiting the p53–MDM2 interaction.[367] High-throughput screening of synthetic chemical libraries provided lead structures that were further developed into the Nutlins. They are highly potent and selective compounds that bind to MDM2 through the p53 binding site. Their rigid scaffold allows presentation of substituents in a way that efficiently mimics p53 binding (Figure 16 d). Some members of the Nutlin family induce cell-cycle arrest and apoptosis in cancer cells in a p53-dependent manner. Moreover, they also inhibit tumor growth in human xenograft models.[368]

Foldamers are validated scaffolds for the design of class B mimetics and proved useful for the development of p53–MDM2 inhibitors. The residues making the biggest contribution in the interaction between p53 and MDM2 (Phe19, Trp23, and Leu26) were integrated into the recognition face of a 14-helix β-peptide. The helical structure was constrained by using the electrostatic macrodipole approach to provide micromolar binders.[369–371] Several methods with improved procedures to synthesize and evaluate β-peptides targeting MDM2 have been reported.[372, 373] However, their relatively poor binding affinities suggest that the 14-helix may not reproduce the p53–MDM2 interaction suitably. Moderate improvements in the biological activity were obtained when non-natural side chains were introduced into these β-peptides.[374, 375] Their cellular uptake was increased by conjugation to cell-penetrating peptides[376] and by the introduction of β-homoarginines[377] or side chain to side chain cross-links.[262] HBS α/β-peptides with the αααβ pattern and hot spots kept as α-amino acids provided affine MDM2 binders with enhanced conformational rigidity.[378] Rationally designed achiral peptoids, preferable with high conformational flexibility, display moderate inhibitory activity of the p53–MDM2 complex.[379]

Structural mimetics (class C) were recently used to inhibit the interaction between p53 and MDM2. Among these mimetics, sterically enforced terphenyls (**27**) with aliphatic groups at the termini and large aromatic substituents at the central position were used to mimic the binding epitope of p53. These mimetics exhibit highest affinity for MDM2 and the best selectivity when binding between MMD2 and BCL-2 family proteins was compared.[380] Notably, these compounds also proved to be active in cell-based assays.[381] Cell-permeable pyrrolopyrimidines (**30**) were also used to disrupt both the p53–MDM2 and the p53–MDMX complexes, thereby promoting p53-dependent apoptosis in cultured cancer cells.[288] Although less potent, hydrogen-bond-guided 3-O-alkylated (**32**),[297, 382] 2-O-alkylated (**33**),[293] and N-alkylated oligobenzamides (**34**)[294, 298, 299] as well as hybrids[300] inhibit the p53–HMD2 complex in vitro. Covalently constrained OHMs (**37**) were also able to bind MDM2 in vitro[383] and the spiroligomers (**38**) disrupted the p53–HDM2 complex and, surprisingly, trigger HDM2 accumulation in cells, probably by preventing proteolytic degradation.[311]

#### 3.2.2. BCL-2 Family Proteins

**Figure 17:**
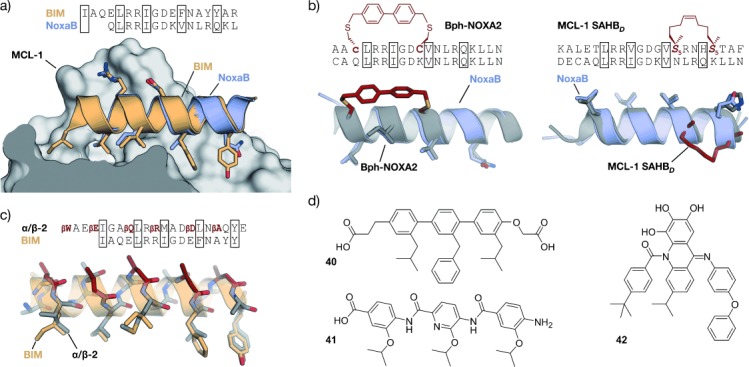
PPIs involving proteins of the BCL-2 family: a) Superimposed crystal structures of BIM (orange, PDB 2L9) and NoxaB (blue, PDB 2NLA) bound to MCL-1.[386] b) Superimposed crystal structures of NoxaB (blue, PDB 2NLA) with (left) bisaryl cross-linked peptide Bph-Noxa2 (gray, PDB 4G35, c=d-cysteine)[204] and (right) stapled peptide MCL-1 SAHB_*D*_ (gray, PDB 3MK8).[387] Cross-links are highlighted in red (side chains of amino acids in boxes are shown explicitly). c) Superimposed crystal structures of BIM (orange, PDB 2L9) and α/β-peptide α/β-2 (gray/red, PDB 4BPI).[388] β-Amino acids are highlighted in red (βE, βQ, βR, βD, and βA are β^3^-amino acids that correspond to E, Q, R, D, and A, respectively). d) Structural mimetics of helical MCL-1 binding peptides.[312, 389, 390]

Proteins of the BCL-2 family play a key role in apoptosis regulation. Both pro-apoptotic (e.g. BAK, BAX, BID, BIM, NOXA, HRK, PUMA, BAD) and anti-apoptotic (e.g. BCL-xL, BCL-2, BCL-w, MCL-1, A1) members of the BCL-2 family participate in a complex network of PPIs.[384, 385] Interactions between members of both classes are involved in the sensing of cellular stress, thereby modulating apoptotic pathways. Pro-apoptotic proteins are classified into effectors, direct activators, and de-repressors or sensitizers. Both effectors (e.g. BAK, BAX) and anti-apoptotic proteins have four BCL-2 homology domains (BH1–BH4) with a shared folding motif that creates a hydrophobic groove, the BC groove. This groove mediates binding to an α-helical stretch of BH3-only proteins, including direct activators (BID, BIM, PUMA) and de-repressors/sensitizers (BAD, NOXA, HRK). This interaction involves highly conserved hydrophobic and polar residues that tightly interact with the BC groove (Figure 17 a). Variations in the remaining BH3 sequence provide the specificity required to precisely orchestrate the interactions within the BCL-2 family members.[384, 385] Proteins of the BCL-2 family are considered targets of high interest in drug development, and their modulation has been addressed by different approaches. Thus, relevant PPIs between BCL-2 family members have been inhibited by class A peptidomimetics, for example, by peptides stabilized by thiol-based cross-links, hydrocarbon-stapling approaches, and by hydrogen-bond surrogates. In addition, class B mimetics such as α/β-peptides, and sterically constrained as well as hydrogen-bond-guided structural (class C) mimetics have been used as inhibitors of these interactions.

By using a bisaryl moiety for the cross-linking of d-Cys (c) and l-Cys at postions *i* and *i*+7, respectively, a NOXA-derived peptide was stabilized to provide a selective binder of MCL-1. The crystal structure of this peptide in complex with MCL-1 (Figure 17 b) revealed the presence of edge-to-face π–π interactions between the aryl cross-link and MCL-1.[204] Using this structure as the basis for further modifications aimed at improving the cellular activity, the hydrophobicity was increased by replacing non-interacting charged amino acids with Ala and by the introduction of backbone N-methylation. A number of “stabilized α-helices of BCL-2 domains” (SAHBs) have been synthesized by applying the peptide-stapling technique. The introduction of the hydrocarbon cross-link increased the helicity, resistance to proteolysis, and cellular uptake of these BH3-derived peptides. However, only some of the peptides were efficient in inhibiting PPIs between BCL-2 family members. Thus, a SAHB from the BH3 domain of the BID protein proved to induce apoptosis in leukemia cells in vitro and in vivo.[391] An MCL-1-derived SAHB inhibits the formation of the BAK-MCL-1 complex, thereby inducing cell death by caspase-dependent apoptosis. The crystal structure of this stapled peptide in complex with MCL-1 (Figure 17 b) proved the direct participation of the staple in target binding. Notably, synthesis and testing of several stapled peptides was required to obtain efficient PPIs inhibitors.[387] SAHB peptides also represent helpful tools that provide valuable insights into the molecular regulation of proteins of the BCL-2 family.[392, 393] Hydrogen-bond surrogates were used to stabilize the BAK BH3 helix, thereby increasing the helicity and proteolysis resistance, although with a loss of binding affinity compared to the wild-type peptide. A subsequent sequence optimization provided a peptide with improved affinity.[394]

Class B peptidomimetics were also used to disrupt PPIs between proteins of the BCL-2 family. Whereas pure β-peptides did not inhibit these PPIs, heterogeneous (e.g. α/β peptides) and chimeric foldamers (e.g. α/β+α) provided the desired inhibitors. The ααβαααβ backbone was applied to a PUMA BH3 derived peptide to obtain foldamers with high binding affinity for BCL-xL and MCL-1 proteins,[395] while the αααβ pattern was used to mimic the BIM BH3 helix and provide binders of the same two proteins.[396] Interestingly, in both cases, the selectivity for the targets is highly dependent on the number and location of α-to-β^3^ replacements. Furthermore, a chimeric peptide (α/β+α) with a 6-mer α-peptide at the C-terminus and a 9-mer α/β-peptide at the N-terminus was ten times more potent than the natural BAK 16-mer and efficiently inhibited formation of the BAK-BCL-xL complex by binding the same cleft targeted by the natural peptide.[397] The N-terminal fragment features alternate 1:1 α- and β-amino acids and displays a new helical arrangement named 14/15 helix.[269] An increase in the proteolytic stability and selectivity within the BCL-2 family members was observed, and release of cytochrome c was confirmed in cell lysates. Subsequent optimization provided foldamers with improved proteolytic stability but negligible cellular uptake.[398, 399]

Terphenyls, as the prototype of class C peptidomimetics, were used to mimic the location of hot-spot residues of helical BH3 peptides.[400, 401] Some of the resulting terphenyls (**40**) disrupt the interaction of BCL-xL, BCL-2, and MCL-1 with BAX or BAK, or with BIM or BAD in cultured cells. This inhibition triggers apoptosis in a caspase-dependent manner.[389] Some pyridazine-containing heterocyclic scaffolds also inhibited the formation of the BAK–BCL-xL complex in vitro.[402] Hydrogen-bond-guided scaffolds such as trispyridylamides,[289] benzoylureas,[305, 403] and biphenyls[302] were also reported to inhibit BAK–BCL-xL complex formation in vitro. The evaluation of oligoamide scaffolds combining different ratios of pyridine and phenyl rings evidenced that the molecules with a higher percentage of phenyl rings disrupt the BAK–BCL-xL complex more efficiently, probably because of their increased hydrophobicity and flexibility. This trend was not translated into increased activity in cell-based assays, which was explained by differences in cell permeability and potential off-target effects.[404] One of these scaffolds with two phenyl rings and one pyridine ring was studied extensively. It mediates apoptosis in cancer cell lines by inhibiting the formation of BAK–BCL-xL and BAK–MCL-1 complexes. Additionally, the compound exhibits inhibitory effects on tumor growth in mouse models (**41**).[390] Notably, terephthalamides also disrupted the BAK–BCL-xL complex formation in human cell culture.[304, 405] NMR spectroscopy and computational studies proved binding to the same cleft as the BAK BH3 peptide. Finally, BIM–MCL-1 and BIM–BCL-2 PPIs were addressed using cross-acridine scaffolds (**42**).[312]

### 3.3. Small GTPases

Small GTPases are switchlike proteins that exist in two distinct conformational states that are defined by their binding to guanosine diphosphate (GDP) or triphosphate (GTP).[406] When bound to GTP, they adopt an active conformation that is capable of binding to effector proteins, thereby triggering downstream signaling events. The nucleotide binding state is regulated by PPIs with guanine nucleotide exchange factors (GEF), which mediate a GDP to GTP exchange, or by GTPase-activating proteins (GAP), which promote hydrolysis of bound GTP to GDP. Malfunctioning of GTPase regulation has implications in numerous human diseases, in particular in cancer formation and propagation. A prime example is the proto-oncogene Ras, which gives its name to a subfamily of related proteins, such as Rab (Ras-related in brain) and Rho (Ras homology) proteins.[407] Their targeting has proved extremely difficult because of the involvement of numerous PPIs in small GTPase regulation and signal propagation.[408] A successful example involves the use of an HBS-stabilized α-helix derived from a GEF protein of Ras (Sos). This modified peptide HBS3 binds the GDP-bound form of Ras with micromolar affinity and is capable of inhibiting the nucleotide exchange by Sos in vitro and in cell culture.[409] Hydrocarbon peptide stapling was used to stabilize an α-helix of the Rab6-interacting protein, an effector of Rab GTPases. Most strikingly, *i*, *i*+4 stapled peptide StRIP3 showed micromolar affinity for the active form of Rab8a and was able to compete with effector binding in vitro.[407] In addition to these class A mimetics, Hamilton and co-workers reported a class C mimetic based on a 5-6-5 imidazole-phenyl-triazole scaffold to target Cdc42, a member of the Rho GTPase family. By mimicking three residues (Leu, Lys, Gln) of the GEF protein Dbs, the compound was able to inhibit the Dbs-promoted nucleotide exchange in vitro (IC_50_=67 μm).[287] However, despite extensive efforts, clinically relevant compounds that directly target small GTPases have not yet been identified.

### 3.4. Transcriptional Regulation

Selective modulation of transcription by designed molecules is very challenging. One reason is the generally frequent involvement of protein–protein interactions in transcriptional regulation. Prime examples are developmental pathways including the NOTCH, Wnt, and Hedgehog signaling cascades. Hyperactivation of such pathways has strong implications in the onset and progression of various types of cancer.[410] A ground-breaking example of the direct targeting of a transcription factor complex using peptidomimetics was reported by the research groups of Verdine and Bradner.[411] They described the design of hydrocarbon-stapled peptides for the inhibition of NOTCH signaling. The activation of NOTCH target genes is facilitated by the binding of protein ligands to NOTCH transmembrane receptors, which triggers proteolytic cleavage of the intracellular domain of NOTCH (ICN).[412] ICN translocates to the nucleus where it activates transcription by forming a trimeric complex with the DNA-bound transcription factor CSL and coactivator proteins of the mastermind-like (MAML) family. By using the α-helical binding domain of MAML as a precursor, the *i*, *i*+4 stapled peptide SAHM1 was designed and showed potent inhibition of trimer formation in vitro and robust cellular uptake.[411] Cell-based assays confirmed the inhibition of NOTCH-dependent gene expression. In a mouse model of NOTCH-driven T-cell acute lymphoblastic leukemia, SAHM1 treatment showed specific antiproliferative effects.[411] Hydrocarbon-stapled peptides were also used to target the Wnt signaling cascade.[222, 234, 235, 413, 414] Canonical Wnt signaling is activated by the binding of extracellular Wnt protein ligands to a receptor complex, which results in intracellular inhibition of a multiprotein destruction complex consisting of scaffolding proteins such as Axin and protein kinases. In the absence of Wnt ligand, this complex is responsible for the degradation of the protein β-catenin. The inhibition of the destruction complex in the presence of Wnt ligand triggers accumulation of β-catenin and its translocation into the nucleus. Here it binds to transcription factors of the LEF/TCF family and coactivators such as B-cell CLL/lymphoma 9 protein (BCL9), thereby activating transcription of Wnt target genes.[410] A direct targeting of β-catenin has been a long standing goal in ligand discovery efforts.[413] Based on the α-helical β-catenin binding epitopes of Axin and BCL9, the *i*, *i*+4 stapled peptides StAx-35R[234] and SAH-BCL9_B_[414] were designed. StAx-35R prevents formation of a complex between β-catenin and LEF/TCF transcription factors, thereby inhibiting target genes under the control of canonical Wnt signaling in cell-based assays.[234] It was shown that the correct subcellular localization is essential for efficient inhibition of the signaling cascade.[235] SAH-BCL9_B_ on the other hand proved effective in targeting the interaction between β-catenin and coactivator BCL9, thereby inhibiting a subset of Wnt target genes that was reported to control stem-cell-like behavior in some forms of cancer. SAH-BCL9_B_ reduced tumor growth, metathesis, and invasion in mouse xenograft models.[414] Notably, hydrocarbon-stapled peptides were also used to modulate other aspects of gene expression. The complex between EZH2 (enhancer of zeste homologue 2) and EED (embryonic ectoderm development and suppressor of zeste 12 homologue) is crucial in histone methylation processes and was inhibited by EZH2-derived stapled peptides.[415] In addition, protein–protein complexes involved in DNA protection mechanisms[416] and in the regulation of mRNA transcription have been targeted using stapled peptides.[87]

**Figure 18:**
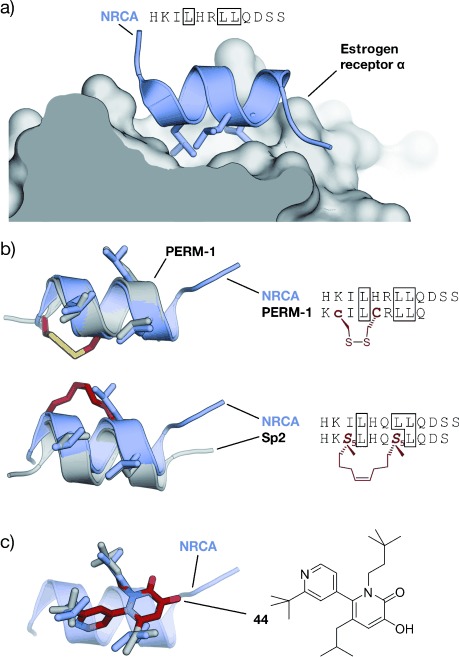
Estrogen receptor (ER) coactivator interaction: a) Coactivator peptide NRCA bound to ER α (gray; PDB 2QGT); b) top: superimposed crystal structures of NRCA (blue, PDB 2QGT) and disulfide cross-linked PERM-1 (gray, PDB 1PCG; left). Cys and d-Cys (c) are highlighted in red, the disulfide bridge in yellow; sequences of cross-linked peptide (right). Bottom: Superimposed crystal structures of NRCA (blue, PDB 2QGT) and stapled peptide Sp2 (gray, PDB 2YJA; left). The cross-link is highlighted in red. Sequences of stapled peptide (right). Selected side chains are shown explicitly and highlighted in sequence. c) Superimposed crystal structures of NRCA (blue, PDB 2QGT) and 6-(2-*tert*-butyl-4-pyridyl)-3-hydroxy-5-isobutyl-1-(3,3-dimethylbutyl)1*H*-pyridin-2-one (44, gray/red, CCDC: 636896).

Estrogen receptors are transcription factors that are activated by steroid hormones and additionally regulated by coactivator proteins. Their hyperactivation has been implicated in several diseases, in particular in the development of cancer.[417] Coactivator proteins bind the receptor through a so-called nuclear receptor box (NR-box) consisting of a LXXLL motif which adopts an α-helical secondary structure upon binding (Figure 18 a). Early approaches to stabilize the binding motif in its active conformation were attempted by introducing disulfide (PERM-1, Figure 18 b), lactam, or thioether side chain to side chain cross-links.[199–201] A lactam-cross-linked peptide was further modified by introducing unnatural amino acids, thereby increasing the selectivity between receptor subtypes.[418] *i*, *i*+4 Stapled peptides were developed based on the crystal structure of nuclear receptor coactivator (NRCA) peptide 2 bound to ERα (Figure 18 a). A series of peptides with various staple positions were investigated in detail.[419] Structural studies revealed significant differences in the binding mode, affinity, and selectivity. Notably, it was possible to replace one of the crucial Leu amino acids by a building block involved in the formation of the marcocycle (Sp2; Figure 18 b). In this case, the hydrophobic cross-link is involved in the binding, thereby leaving the remaining residues of the stabilized peptide in good alignment with the wild-type peptide.[419] Hamilton and co-workers introduced a structural mimetic based on pyridylpyridone derivatives with substitutions in the 2-pyridyl and 1,5-pyridone positions (e.g. **44**) to provide compounds that show competition with the natural binding sequence in vitro. The crystal structure of unbound **44** reveals a good alignment of its hydrophobic substituents with the Leu side chains of the helical LXXLL motif (Figure 18 c).[286]

The interaction between hypoxia-inducible transcription factors (HIFs) and p300/CBP coactivator proteins is another PPI with implications in the occurrence of cancer. HIFs are expressed under hypoxia, the cellular state of reduced oxygen levels. In cancer cells, the interaction with its coactivators can trigger the expression of genes that promote invasion, angiogenesis, and a modified metabolism.[420] The interaction between HIF-1α and p300/CBP is mediated by two short α-helices in HIF-1α. Based on these peptide sequences, Arora and co-workers designed a number of different peptidomimetics. Initial efforts focused on hydrogen-bond surrogates (**26**) to yield modified peptides that potently inhibit complex formation.[246, 421] Notably, the stabilized peptides showed inhibitory effects both in cancer-cell-based assays and murine tumor xenografts. In addition, they tested class C peptidomimetics for their potential to inhibit the HIF-1α-p300/CBP interaction.[383, 422, 423] Aromatic oligoamides (**32** and **34**) were used to project three aliphatic side chains of the HIF-1α sequence and showed inhibitory effects in vitro.[423] Oligooxopiperazine helix mimetics (OHM, **37**) were also used to align not only aliphatic but also polar residues.[383, 422] The resulting class C mimetic OHM-1 does not only compete with HIF-1α binding in vitro, but also reduces the expression of hypoxia-inducible genes in cell-based assays and is active in murine tumor xenografts. These results underline the remarkable potential of α-helix mimetics based on the oligooxopiperazine scaffold.

### 3.5. Enzyme Regulation

Targeting of enzymes through their active sites or cofactor binding sites is one of the prime applications of small molecular inhibitors. However, in many cases, PPIs are involved in the regulation of enzyme function through altering enzyme reactivity or subcellular localization, thus suggesting the use of peptidomimetics for their modulation. Often, conserved protein domains such as SRC homology 3 (SH3), PDZ, and WW are involved in these regulatory PPIs.[424] Such domains recognize certain peptide stretches within flexible regions of their protein-binding partners. SH3 domains, for example, bind to PXXP sequences, with X representing variable amino acids that mediate subtype specificity.[424] It has been shown that SH3 domains specifically bind proline because of its N-substitution, and not because of the cyclic nature or bulkiness. Thus, the incorporation of N-alkylated moieties in a peptide sequence resulted in peptoid–peptide hybrids that proved to be potent and highly selective ligands of SH3 domains.[275] The subcellular localization of protein kinase A (PKA), a key protein in the regulation of signaling pathways involving cyclic AMP, is determined by A-kinase anchoring proteins (AKAPs). These PPIs are mediated by helical peptide structures that have been used as a starting point for the design of stapled peptides. These class A mimetics exhibit high proteolytic stability, good cell permeability, and isoform specificity, thereby allowing selective inhibition of PKA-RII-AKAP complex formation in vitro and in cultured cells.[425] Piperidine–piperidinone-based molecules (**18**) were used as structural mimetics of β-strands involved in the homodimerization of α-antithrombin.[426] These class C mimetics have the ability to perturb dimerization, thereby catalyzing α-antithrombin oligomerization. This is a unique example of an application of structural β-strand mimetics that points towards the so far unexplored potential of these scaffolds.

### 3.6. Pathogenic Targets

Infectious diseases represent the second leading cause of death worldwide.[427] They are caused by pathogenic microorganisms such as viruses, bacteria, fungi, or parasites. The life cycle of these pathogens involves numerous PPIs which are crucial for essential functions such as host recognition, reproduction, and defense strategies. Thus, the development of compounds that selectively inhibit pathogenic PPIs represents a promising therapeutic strategy. A number of structure-based design strategies have been used to develop PPI inhibitors that combat certain aspects of viral or bacterial infections. The human immunodeficiency virus (HIV) represents a prime target for the application of a wide range of peptide-derived PPI inhibitors, with a focus on targeting virus assembly and its entry into the host cell. A key process in HIV infection is the assembly of both immature-like viral particles first and, after proteolytic cleavage, mature viral capsids.[428] Phage-display screenings provided a helical peptide sequence (CAI) that disrupts the assembly of these particles in vitro, without exhibiting sufficient cell permeability.[429] An application of the hydrocarbon stapling technique resulted in stapled peptide NYAD-1, which shows increased α-helicity and binding affinity in vitro. NYAD-1 is able to penetrate cells and inhibit HIV infection in cell-based assays.[430] However, low solubility and self-association issues have been detected for NYAD-1 and analogues.[431]

**Figure 19:**
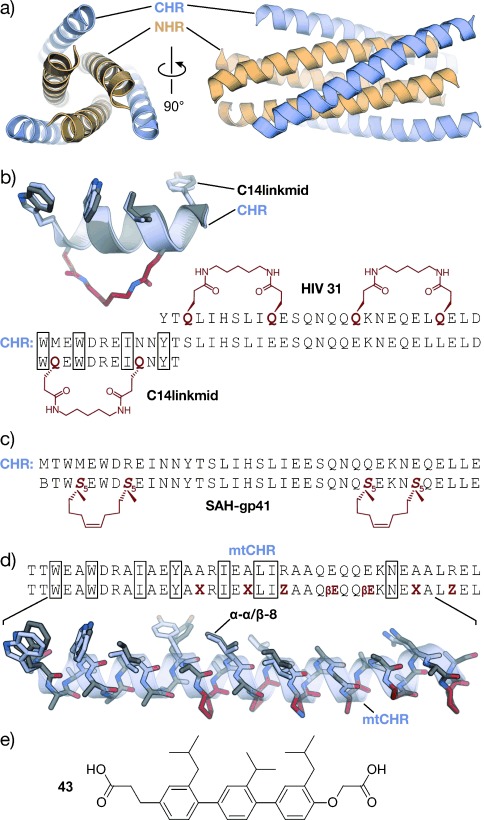
Six-helix bundle of gp-41 CHR and NHR helices: a) Crystal structure of the six-helix bundle involving three CHR (blue) and three NHR (orange) helixes (PDB 1AIK).[432] b) Two examples of lactam-bridged stabilized CHR-derived α-helices: HIV 31[434] and C14Linkmid including superimposed crystal structures of a CHR fragment (blue, PDB 1AIK) and C14Linkmid (gray, PDB 1GZL).[216] The amide cross-link is highlighted in red. c) Sequence of CHR-derived double-stapled peptide SAH-gp41.[232] d) Chimeric peptide α-α/β-8 derived from a mutant CHR form (mtCHR). Superimposed crystal structures of mtCHR (blue, PDB 3F4Y) and α-α/β-8 (gray, PDB 3G7A).[439] β-Amino acids are highlighted in red (X=ACPC, Z=APC, βE=β^3^-glutamate). e) CHR-derived terphenyl structural mimetic[280] (side chains of amino acids in boxes are shown explicitly in the crystal structures).

Glycoprotein 41 (gp41) is an HIV transmembrane protein responsible for the fusion of the viral envelope and host cell membrane, thus rendering gp41 a valuable therapeutic target. The fusion process involves the insertion of the N-terminal coiled coil of gp41 into the host cell followed by a structural rearrangement, which results in the formation of a six α-helix bundle. This rearrangement brings the viral envelope and host membrane in proximity and leads to their fusion.[432] The six α-helix bundle (Figure 19 a) comprises three inner α-helices originating from the N-terminal heptad repeat of gp41 (NHR, orange), and three outer α-helices from the C-terminal heptad repeat (CHR; blue Figure 19). The FDA approved drug Enfuvirtide is a 36-amino acid unmodified peptide derived from CHR.[433] Enfuvirtide proved the general applicability of peptides as antiviral therapeutics and fostered the development of various class A mimetics that target the assembly of the helix bundle. A series of CHR-derived peptides were stabilized by the introduction of one (e.g. HIV C14 Linkmid)[216] or two lactam cross-links (e.g. HIV31, Figure 19 b).[434] In cell-based assays, the most active peptides in these series exhibit micromolar inhibitory activities against viral infection. The CHR sequence also served as a template for peptides stabilized by means of hydrogen-bond surrogates (HBS). After improving the solubility of these peptides by incorporation of basic and acidic residues at non-interacting positions, they displayed micromolar inhibition of cell fusion.[435] The peptide sequence of Enfuvirtide was used as a starting point for the design of a stapled peptide containing two all-hydrocarbon cross-links. The double-stapled version of Enfuvirtide (SAH-gp41, Figure 19 c) features a highly α-helical character and exhibits increased resistance to proteolysis, oral bioavailability, and HIV-1 fusion inhibitory activity when compared to the unmodified peptide.[232]

Short β-peptides featuring hydrophobic residues of the CHR peptide were also designed to inhibit the formation of the six-helix bundle. These class B mimetics adopt a 14-helical conformation which is assisted by electrostatic stabilization of the macrodipole and salt bridges between ornithine and glutamic acid side chains placed in one single face of the helix.[436] An improvement of bundle disruption was obtained after optimization of the central tryptophan residue.[437] The design of CHR-derived long heterogeneous α/β-peptides resulted in helical foldamers. The use of the ααβαααβ pattern afforded foldamers that align all the β-residues on the solvent-exposed face of the helix.[438] The large number of β-amino acids renders these foldamers relatively flexible, which translates into a significant loss of inhibitory activity. Chimeric peptides combining a stretch purely composed of α-amino acids with an α/β-region showed enhanced binding to the NHR, but again with reduced proteolytic stability. Notably, the replacement of standard β-amino acids by cyclic ones increased the helicity, thereby promoting proteolytic stability, binding affinity, and antiviral activity (α-α/β-8, Figure 19 d).[439] In addition, it was shown that the introduction of adjacent acidic and basic β-amino acids can enhance the helicity.[440] Structural mimetics were also used to target the gp41 protein. Terphenyls containing liphophilic substituents (**43**) such as branched aliphatic groups or benzyl rings were designed to target NHR peptides, thereby inhibiting bundle formation and cell fusion in cell-based assays.[280] Chemokine receptor subtype CXCR4 is a human GPCR that represents one of the major co-receptors involved in the entry of HIV into the host cell.[441] Natural disulfide-bridged β-hairpins of the Tachyplesin family and their numerous analogues belong to the first examples of inhibitors of CXCR4-mediated HIV entry.[137–140] The simplification of these β-hairpin structures has provided very active head-to-tail cyclic pentapeptides,[141] which were further improved by installation of conformational constraint through incorporation of peptoid monomers.[276]

The fusion mechanism exhibited by HIV is highly conserved among related viruses, including the respiratory syncytial virus (RSV). The F1 glycoprotein is the transmembrane protein responsible for RSV fusion with host cells. In analogy to anti-HIV strategies, the entry of RSV into cells has also been blocked with peptides inspired by the HRC region of this F1 glycoprotein. These α-helical peptides were stabilized using two lactam cross-links and show inhibitory activity against RSV fusion.[217, 442] Similarly, double-stapled peptides also proved useful for inhibiting RSV infection. Notably, intranasal administration of these peptides and conjugation with nanoparticles resulted in in vivo activity.[443] Entry of the hepatitis C virus (HCV) into host cells is partially mediated by the interaction of the viral envelope glycoprotein E2 with the human CD81 receptor.[444, 445] One of the two extracellular loops of CD81 served as the inspiration for the design of a linear peptide that weakly disrupts the interaction between CD81 and HCV E2.[446] An *i*, *i*+7 stapled version of this peptide showed increased α-helicity and proteolytic stability, accompanied with a significant enhancement in inhibiting the HCV entry.[447]

**Figure 20:**
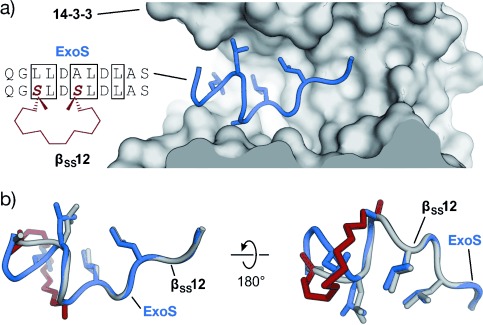
Interaction between 14-3-3 and ExoS: a) crystal structure of the 14-3-3 binding sequence of exoenzyme S (blue, ExoS) in complex with 14-3-3 (gray, PDB 4N7G). Sequences of ExoS and corresponding cyclic peptide inhibitor β_SS_12 are shown (side chains of amino acids in boxes are shown explicitly in the crystal structures). b) Overlaid structures of ExoS (blue, PDB 4N7G) and cyclic peptide β_SS_12 (gray, PDB 4N84). The cross-link is highlighted in red.[92]

The Gram-negative bacterium *Pseudomonas aeruginosa* secrets virulence factors into the host cytosol, thereby manipulating various signaling pathways. One of these factors is the protein exoenzyme S (ExoS). ExoS requires the formation of a complex with the human protein 14-3-3 to implement some of its pathogenic effects. The ExoS–14-3-3 interaction is mediated by a central 11-mer peptide sequence of ExoS binding the globular domain of 14-3-3 (Figure 20 a). This 11-mer peptide adopts a structure composed of several overlapping turns with five hydrophobic residues binding to a lipophilic patch on 14-3-3.[448] Replacement of two of these hydrophobic residues by a hydrocarbon cross-link was used to design a series of cross-linked peptides.[92] The most affine peptide β_SS_12 (Figure 20 a) showed a 20-fold increased affinity towards 14-3-3 compared to the unmodified sequence. The crystal structure of β_SS_12 in complex with 14-3-3 reveals a configuration of the backbone that is very similar to the initial turn motif (Figure 20 b), thereby representing the first example of an artificially stabilized turn motif. Notably, β_SS_12 is capable of inhibiting the interaction between a fragment of ExoS and 14-3-3 in vitro.

## 4. Conclusion

The discovery of molecules that selectively bind to a protein target is in many cases a challenging endeavor. Often, the screening of large compound collections with biochemical or cell-based assays provides valuable hit structures that can be evolved in an iterative process. However, the identification of selective PPI inhibitors through the screening of classic small-molecule libraries proves to be particularly difficult due to the different structural requirements of protein–protein interfaces. The rational design of inhibitors using peptide binding epitopes derived from protein–protein complexes represents an alternative approach. The enormous structural information contained in the protein data bank[186] provides an extremely valuable source for the structure-based design of novel ligands. As a consequence of their flexible nature in the unbound state, unmodified short peptide sequences often exhibit reduced target affinity and low proteolytic stability as well as cell permeability, thus limiting their use as biologically active agents. In addition, the structural context of a binding epitope (e.g. embedded in globular domain or flexible region) can affect the binding characteristics of the isolated peptide. By mimicking the bioactive conformation of the peptide, peptidomimetics aim to improve the binding affinity and bioavailability. This Review offers a comprehensive overview of structure-based approaches towards the development of peptidomimetics that are used as PPI inhibitors. All types of secondary structure elements—turns, β-sheets, and helices—have been identified as PPI recognition motifs,[36] thus rendering mimetics of these structures potentially valuable for PPI inhibition. Interestingly, the overall spatial arrangement of secondary structure elements in PPI interfaces appear to be limited,[449] which allows certain secondary structure mimics to be used for different targets. Binding selectivity is then determined by the nature of the corresponding side chain mimicking substituents. This concept has been successfully implemented for repetitive secondary structures, in particular for α-helices. However, a generic approach that could provide a construction manual for irregular turn structures remains elusive.[450]

Reproducing the bioactive conformation of a peptide binding epitope is the first intrinsic obstacle when designing peptidomimetics. Endowing these inhibitors with appropriate pharmacokinetic properties is the second challenge. The balance between these requirements is complex: While a low degree of difference from the parent peptide supports good binding properties, it may hamper bioavailabilty. In contrast, small-molecule scaffolds are more druglike, but less prone to the selective binding of extended protein surfaces. Notably, the rational design of peptidomimetics becomes more challenging as the degree of difference increases. To emphasize the level of difference relative to the parent peptide and to allow a clear assignment of available approaches, we introduce a novel classification of peptidomimetics (classes A–D). Class A mimetics encompass conformationally constrained peptides with moderate modifications that yield peptidomimetics with the highest degree of similarity to their respective parent peptide. Macrocyclization is the key constraining element for all secondary structures. Numerous cross-link architectures have been developed, particularly for α-helices, with most examples of biologically active mimetics involving stapled peptides. Recent comparative studies indicate that various other cyclization approaches can provide helical class A mimetics with comparable improvements in target affinity.[329, 451, 452] However, the effect of these cross-linking strategies on the bio-availability of the resulting mimetics still has to be evaluated in detail. Further modification of class A mimetics, or the grafting of interacting peptide residues on foldamers, result in class B mimetics, which proved to be particularly resistant to proteolytic degradation. As a consequence of the rather high degree of difference to the parent peptide, foldamers frequently exhibit reduced inhibitory activity, but additional optimization efforts can compensate this initial drawback. Although some class B mimetics exhibit higher cell permeability than their peptide analogues, a general investigation of this feature is lacking. In class C mimetics (structural mimetics), the entire peptide backbone is replaced by a small molecular scaffold. This fundamental change and the fact that often only a few of the epitope residues are mimicked render the design process particularly demanding. In many cases, this requires additional efforts to re-install selective target recognition; however, further optimization of these mimetics is in some cases hampered by their limited synthetic access. Only a very small number of class C PPI inhibitors has been tested in complex disease models, thus making an evaluation of their pharmacological properties difficult. In the PPI context, most class C examples involve helix mimetics, thus revealing the need for suitable scaffolds that mimic β-strand and turn structures. For turns, numerous class A and C mimetics have been described to inhibit enzymes and peptide–protein interactions, thus also suggesting a use of these approaches for the design of PPI inhibitors in the future. Notably, class D mimetics (mechanistic mimetics) identified in screening approaches can be expected to provide more PPI inhibitors in the future, as novel and structurally more diverse compound libraries have been developed that are more suitable for targeting extended protein surfaces.

The design process of peptidomimetics usually involves an initial major alteration of the peptide backbone, thereby resulting in class A, B, or C mimetics. A subsequent and iterative optimization can follow that aims for improved affinity and bioavailability. However, very rarely do these modifications result in peptidomimetics of the next higher class, as observed for some class A helix mimetics that have been converted into class B mimetics.[365] Overall, the second iterative step is rarely pursued, thus leaving the full potential of some peptidomimetics unexplored. To some extent this is caused by the difficult synthetic access, particularly of class C mimetics. Thus, simplification of this optimization process and transfer of the desired properties between classes is desirable and can be expected to provide PPI inhibitors with improved biological activity. However, general conclusions have to be made with caution, as neither the secondary structures nor the classes of peptidomimetics have been investigated with equal intensity. Notably, class A helix mimetics represent the most evolved group of peptidomimetics, with the largest number of examples of biologically active inhibitors. This may distort the comparison of different mimicking approaches. Importantly, certain scaffolds have the potential to mimic more than one type of secondary structure (e.g. helices and β-hairpins,[115] or helices and certain turn structures[86]), which may expand the use of already evolved peptidomimetics.

Recently, the use of peptide-based drugs has experienced a renaissance, which is highlighted by six peptides being approved as novel drugs in 2012.[453] This trend is supported by the development of enhanced drug-delivery systems.[454] Given the limitations of unmodified peptides, this is a remarkable trend that indicates the potential of peptidomimetics to reduce peptide-associated drawbacks. In this respect, a more detailed understanding of the advantages and limitations of certain mimicry approaches is crucial for their efficient application. An important goal is a coherent set of techniques that can be applied depending on the characteristics of a given target protein. Once this is established, borders between the different classes of peptidomimetics will become more diffuse. A selective targeting of intracellular PPIs with orally available molecules remains elusive and is still one of the ultimate goals in drug development. Recently, this ambitious aim appears to be becoming achievable from the recent progress of peptidomimetics as potent PPI inhibitors and the growing insights into factors that govern their bioavailability.
